# Genome-wide screening reveals the genetic basis of mammalian embryonic eye development

**DOI:** 10.1186/s12915-022-01475-0

**Published:** 2023-02-03

**Authors:** Justine M. Chee, Louise Lanoue, Dave Clary, Kendall Higgins, Lynette Bower, Ann Flenniken, Ruolin Guo, David J. Adams, Fatima Bosch, Robert E. Braun, Steve D. M. Brown, H.-J. Genie Chin, Mary E. Dickinson, Chih-Wei Hsu, Michael Dobbie, Xiang Gao, Sanjeev Galande, Anne Grobler, Jason D. Heaney, Yann Herault, Martin Hrabe de Angelis, Fabio Mammano, Lauryl M. J. Nutter, Helen Parkinson, Chuan Qin, Toshi Shiroishi, Radislav Sedlacek, J-K Seong, Ying Xu, The International Mouse Phenotyping Consortium, Cheryl Ackert-Bicknell, Douglas Adams, Anne-Tounsia Adoum, Juan A. Aguilar-Pimentel, Uchechukwu Akoma, Dalila Ali-Hadji, Oana V. Amarie, Philippe André, Aurelie Auburtin, Chaouki Bam’Hamed, Johannes Beckers, Joachim Beig, Zorana Berberovic, Alexandr Bezginov, Marie-Christine Birling, Katharina Boroviak, Joanna Bottomley, Antje Bürger, Dirk H. Busch, Natalie C. Butterfield, Pilar Cacheiro, Julia Calzada-Wack, Emma L. Cambridge, Susan Camilleri, Marie-France Champy, Heather Cater, Philippe Charles, Elissa J. Chesler, Yi-Li Cho, Audrey E. Christiansen, Valentina Cipriani, Nicola Cockle, Gemma Codner, Amie Creighton, Maribelle Cruz, Katharine F. Curry, Abigail D’Souza, Ozge Danisment, Daniel Delbarre, Hannah F. Dewhurst, Brendan Doe, Alex Dorr, Florian Giesert, Graham Duddy, Kyle Duffin, Amal El Amri, Hillary Elrick, Mohammad Eskandarian, Martin Fray, Anthony Frost, Helmut Fuchs, Valerie Gailus-Durner, Karen K. Gampe, Milan Ganguly, David Gannon, Lillian Garrett, Marina Gertsenstein, Diane Gleeson, Leslie Goodwin, Jochen Graw, Kristin Grimsrud, Hamed Haselimashhadi, Liane Hobson, Andreas Hörlein, Sabine M. Hölter, Seung-Hyun Hong, Neil Horner, Amanda G. Trainor, Ziyue Huang, Coleen Kane, Yulia Katsman, Lance C. Keith, Lois Kelsey, Janet Kenyon, Ruairidh King, Piia Keskivali-Bond, Andrea Kirton, Tanja Klein-Rodewald, Thomas Klopstock, Davide Komla-Ebri, Tomasz Konopka, Ralf Kühn, Fiona Kussy, David Lafont, Qing Lan, Denise G. Lanza, Valerie Laurin, Elise Le Marchand, Sophie Leblanc, Victoria D. Leitch, Chris Lelliott, Christoph Lengger, Lauri Lintott, John G. Logan, Isabel Lorenzo, Ann-Marie Mallon, Naila S. Mannan, Susan Marschall, Melissa L. McElwee, Matthew Mckay, Robbie S. B. McLaren-Jones, Jeremy Mason, Terrence F. Meehan, David Miller, Michayla Moore, Violeta Munoz-Fuentes, Stephen A. Murray, Dong Nguyen-Bresinsky, Oskar Oritz, Panos Pandis, Alexandru Parlog, Amit Patel, Guillaume Pavlovic, Monica Pereira, Kevin Peterson, Vivek Philip, Andrea S. Pollard, Jan Prochazka, Dawei Qu, Ayexa Ramirez, Sean Rangarajan, Tara L. Rasmussen, Birgit Rathkolb, Mike Relac, Kyle Roberton, Willson Roper, Stéphane Rousseau, David W. Rowe, Jan Rozman, Jennifer Ryan, Edward J. Ryder, Luis Santos, Adrián Sanz-Moreno, Joel Schick, Zachary Seavey, John R. Seavitt, Claudia Seisenberger, Mohammed Selloum, Xueyuan Shang, Dong-Guk Shin, Michelle Simon, Gillian Sleep, Damian Smedley, Tania Sorg, Penny C. Sparkes, Nadine Spielmann, Ralph Steinkamp, Michelle Stewart, Claudia Stoeger, Ewan Straiton, Karen L. Svenson, Holly Swash, Lydia Teboul, Sandra Tondat, Irina Treise, Catherine Tudor, Rachel Urban, Valerie E. Vancollie, Laurent Vasseur, Igor Vukobradovic, Hannah Wardle-Jones, Jonathan Warren, Marie Wattenhofer-Donze, Sara E. Wells, Jacqueline K. White, Jean-Paul Wiegand, Amelia Willett, Catherine Witmeyer, Eckhard Wolf, Leeyean Wong, Joshua Wood, Wolfgang Wurst, Catherine Xu, Annemarie Zimprich, Brian Brooks, Colin McKerlie, K. C. Kent Lloyd, Henrik Westerberg, Ala Moshiri

**Affiliations:** 1 Oakland University William Beaumont School of Medicine, Rochester, MI USA; 2 Mouse Biology Program, University of California Davis, Davis, CA USA; 3 University of Miami: Miller School of Medicine, Miami, FL USA; 4 The Centre for Phenogenomics, Toronto, ON Canada; 5 Lunenfeld-Tanenbaum Research Institute, Sinai Health, Toronto, ON Canada; 6 The Hospital for Sick Children, Toronto, ON Canada; 7 The Wellcome Trust Sanger Institute, Wellcome Genome Campus, Hinxton, Cambridge, UK; 8 Centre of Animal Biotechnology and Gene Therapy (CBATEG), Universitat Autònoma de Barcelona, Barcelona, Spain; 9 The Jackson Laboratory, Bar Harbor, ME USA; 10 Medical Research Council Harwell Institute, Mammalian Genetics Unit and Mary Lyon Centre, Harwell Campus, Oxfordshire, UK; 11 National Laboratory Animal Center, National Applied Research Laboratories (NARLabs), Taipei City, Taiwan; 12 Department of Molecular and Human Genetics, Baylor College of Medicine, Houston, TX USA; 13 Phenomics Australia, The John Curtin School of Medical Research, Canberra, Australia; 14 Nanjing Biomedical Research Institute, Nanjing University, Nanjing, China; 15 Indian Institutes of Science Education and Research, Pune, India; 16 Faculty of Health Sciences, PCDDP North-West University, Potchefstroom, South Africa; 17 Institut de Génétique et de Biologie Moléculaire et Cellulaire, Université de Strasbourg, Illkirch, France; 18 German Mouse Clinic, Institute of Experimental Genetics, Helmholtz Zentrum München, Neuherberg, Germany; 19 Monterotondo Mouse Clinic, Italian National Research Council (CNR), Monterotondo Scalo, Italy; 20 European Bioinformatics Institute, Wellcome Genome Campus, Hinxton, Cambridgeshire, UK; 21 National Laboratory Animal Center, National Applied Research Laboratories, Beijing, China; 22 RIKEN BioResource Center, Tsukuba, Japan; 23 Czech Center for Phenogenomics, Institute of Molecular Genetics of the Czech Academy of Sciences, Vestec, Czech Republic; 24 Research Institute for Veterinary Science, College of Veterinary Medicine, Seoul National University, Seoul, South Korea; 25 CAM-SU Genomic Resource Center, Soochow University, Suzhou, China; 26 Ophthalmic Genetics and Visual Function Branch, National Eye Institute, NIH, Bethesda, MD 20892 USA; 27 Department of Laboratory Medicine & Pathobiology, Faculty of Medicine, University of Toronto, Toronto, ON Canada; 28 Department of Surgery, School of Medicine, University of California Davis, Sacramento, CA USA; 29 Department of Ophthalmology & Vision Science, School of Medicine, University of California Davis, Sacramento, CA USA; 30 UC Davis Eye Center, 4860 Y St., Ste. 2400, Sacramento, CA 95817 USA

**Keywords:** MAC spectrum, Eye development, Mouse, IMPC, Serine-glycine biosynthesis, CPLANE

## Abstract

**Background:**

Microphthalmia, anophthalmia, and coloboma (MAC) spectrum disease encompasses a group of eye malformations which play a role in childhood visual impairment. Although the predominant cause of eye malformations is known to be heritable in nature, with 80% of cases displaying loss-of-function mutations in the ocular developmental genes OTX2 or SOX2, the genetic abnormalities underlying the remaining cases of MAC are incompletely understood. This study intended to identify the novel genes and pathways required for early eye development. Additionally, pathways involved in eye formation during embryogenesis are also incompletely understood. This study aims to identify the novel genes and pathways required for early eye development through systematic forward screening of the mammalian genome.

**Results:**

Query of the International Mouse Phenotyping Consortium (IMPC) database (data release 17.0, August 01, 2022) identified 74 unique knockout lines (genes) with genetically associated eye defects in mouse embryos. The vast majority of eye abnormalities were small or absent eyes, findings most relevant to MAC spectrum disease in humans. A literature search showed that 27 of the 74 lines had previously published knockout mouse models, of which only 15 had ocular defects identified in the original publications. These 12 previously published gene knockouts with no reported ocular abnormalities and the 47 unpublished knockouts with ocular abnormalities identified by the IMPC represent 59 genes not previously associated with early eye development in mice. Of these 59, we identified 19 genes with a reported human eye phenotype. Overall, mining of the IMPC data yielded 40 previously unimplicated genes linked to mammalian eye development. Bioinformatic analysis showed that several of the IMPC genes colocalized to several protein anabolic and pluripotency pathways in early eye development. Of note, our analysis suggests that the serine-glycine pathway producing glycine, a mitochondrial one-carbon donator to folate one-carbon metabolism (FOCM), is essential for eye formation.

**Conclusions:**

Using genome-wide phenotype screening of single-gene knockout mouse lines, STRING analysis, and bioinformatic methods, this study identified genes heretofore unassociated with MAC phenotypes providing models to research novel molecular and cellular mechanisms involved in eye development. These findings have the potential to hasten the diagnosis and treatment of this congenital blinding disease.

**Supplementary Information:**

The online version contains supplementary material available at 10.1186/s12915-022-01475-0.

## Background

The molecular mechanisms of microphthalmia, anophthalmia, and coloboma (MAC) spectrum disease are not fully defined. This study searched 8267 single gene knockout mouse lines produced and phenotyped by the International Mouse Phenotyping Consortium [1–4], to identify the novel genes and pathways required for mammalian eye development and MAC spectrum disease.

Eye development begins shortly after gastrulation with the specification of a single eye field in the anterior neural plate by a set of highly conserved eye field transcription factors [5, 6]. During neurulation, two lateral optic pits form as the lateral walls of the diencephalon protrude outward, forming the left and right optic vesicles, and can be visually detected in mice at ~E8.0 [7]. Anteriorly, these optic vesicles come in contact with the surface ectoderm to form the optic placode at ~E9.0, which will develop into the lens and a portion of the cornea. Posteriorly, the neural ectoderm invaginates to form the optic cup and the optic stalk which eventually form the neural retina, the retinal pigmented epithelium (RPE), and the optic nerve. A furrow forms between the optic stalk and the optic cup, forming the choroidal fissure, which eventually fuses together to form the eye [8]. MAC spectrum eye defects can occur at any stage of ocular embryogenesis, with microphthalmia and anophthalmia usually occurring at 4 to 6 weeks of human gestation [9]. While most of these cases occur in isolation within a continuum of phenotypic severity, one-third of cases present as part of a multi-systemic syndrome [10].

Clinically in humans, microphthalmia is defined as having an axial length of less than 10 mm (more than two standard deviations below 21 mm) and a corneal diameter of less than 10 mm. Anophthalmia is characterized as having no ocular tissue within the orbit. There are three different categories of anophthalmia: primary anophthalmia caused by a failure of the optic pit to develop into the optic vesicle, secondary anophthalmia caused by a developmental failure within the anterior neural tube, and degenerative anophthalmia, where the optic vesicles initially form, but eventually degenerate [10]. Coloboma is a closure defect within the optic fissure, typically causing a “keyhole” shape within the iris, inferior lens irregularity, and an inferonasal defect in the retina and choroid layers of the eye, though patients may only exhibit the posterior findings. These eye malformations play a role in childhood visual impairment, with microphthalmia being present in up to 11% of blind children [11–13]. The prevalence of microphthalmia is approximated as one in 7000 live human births, one in 30,000 live births for anophthalmia, and one in 5000 live births for coloboma [14]. The predominant cause of early eye malformations is heritable in nature, and up to 10–15% of siblings of individuals with microphthalmia and/or anophthalmia have a risk of being affected [9]. In severe cases of bilateral microphthalmia or anophthalmia, about 80% of cases show loss-of-function mutations in OTX2 or SOX2 [14]. The genetic abnormalities within the other 20% of patients with MAC spectrum disease are mostly unknown. In addition, the pathways involved in embryogenesis of the eye are also incompletely understood. Therefore, through systematic forward screening of the mammalian genome, this study provides additional insight into the genetic basis of the morphogenetic pathways leading to MAC spectrum diseases, which may ultimately contribute to better detection, diagnosis, and therapy.

## Results

Query of the IMPC phenotype database (August 2022/IMPC data release 17) identified 74 knockout mouse lines with significantly higher incidence of eye anomalies compared to WT (wild-type) controls, suggesting these genes are implicated in embryonic eye development (see Additional file 1: Table S1 for a complete gene list). Genes resulting in eye anomalies were noted at different stages of development: E9.5 (*n* = 8), E12.5 (*n* = 14), E15.5 (*n* = 37), and E18.5 (*n* = 15), typically associated with the windows of lethality of the HOM mutants. However, for 11 of the genes, MAC phenotypes were noted at more than one developmental age. As these genes were embryo lethal, the eye phenotypes were present predominantly in HOM embryos (> 90%). For 27 of these 74 genes, ocular anomalies were noted in HET adult mice during standardized examination of the anterior and posterior segments of the eyes performed at 15 weeks of age as part of the IMPC adult phenotyping pipeline.

**Fig. 1:**
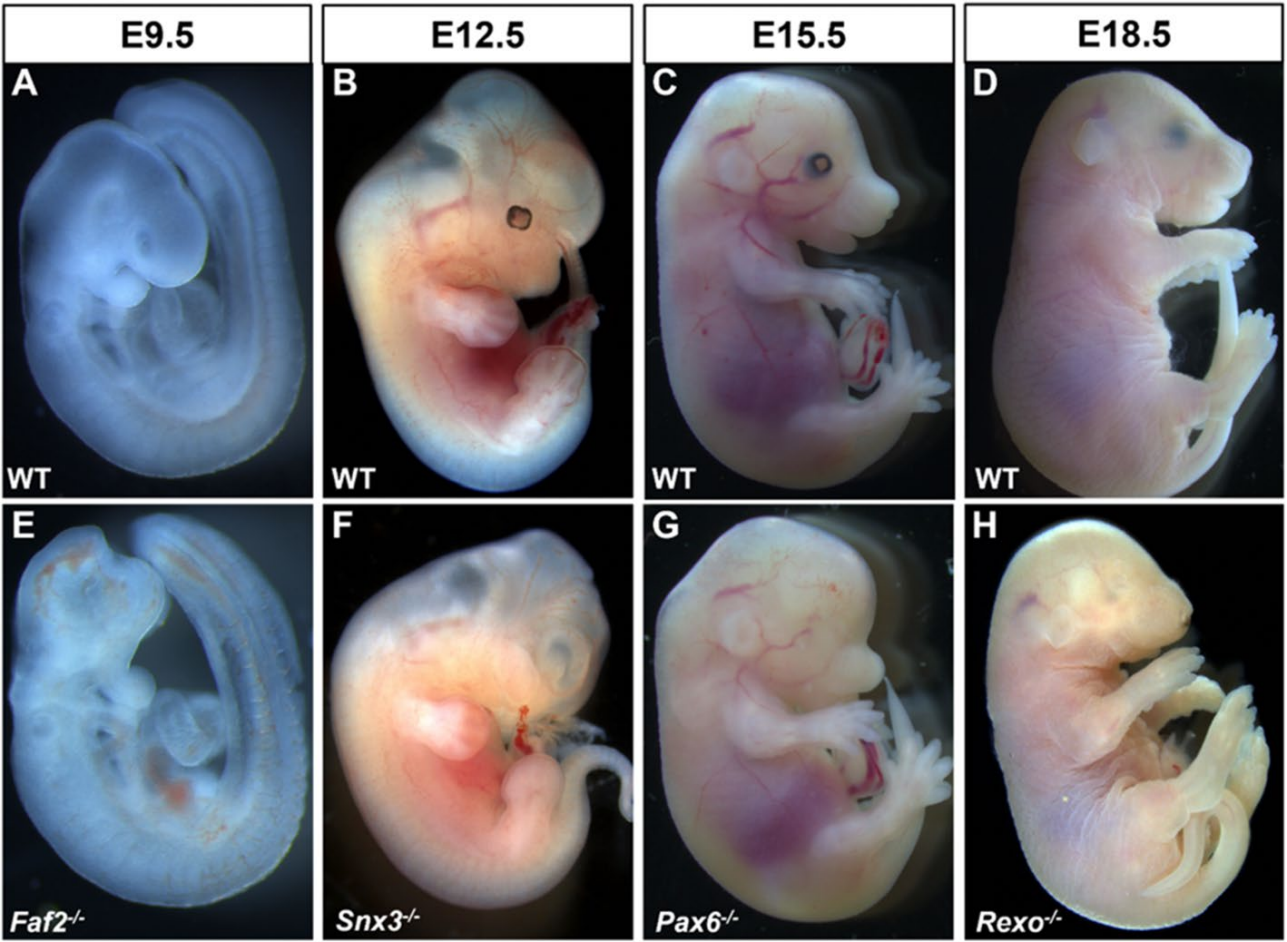
Examples of wild-type (WT) control (**A**–**D**) and mutant embryos (**E**–**H**) with homozygous null mutations of genes associated with anophthalmia at different stages of development. Typically developing mice (WT, **A**–**D**) on C57BL/6N background are shown for comparison

**Fig. 2:**
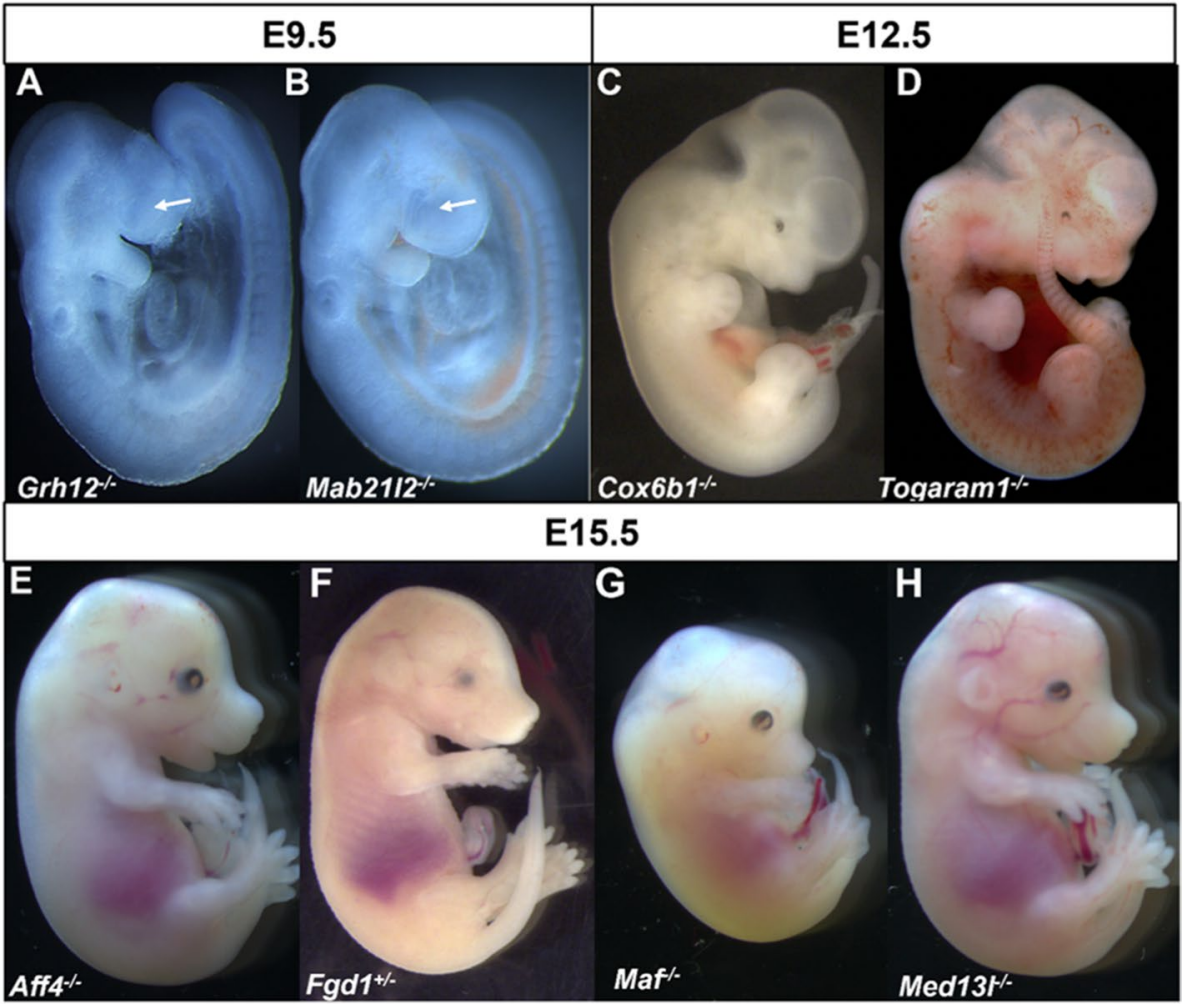
Examples of embryos with homozygous null mutations of genes associated with abnormal development of optic vesicle at E9.5 (arrows, **A**, **B**), microphthalmia and coloboma at E12.5 (**C**, **D**), and E15.5 (**E**–**H**). For typically developing eye, please refer to Fig. 1

**Fig. 3:**
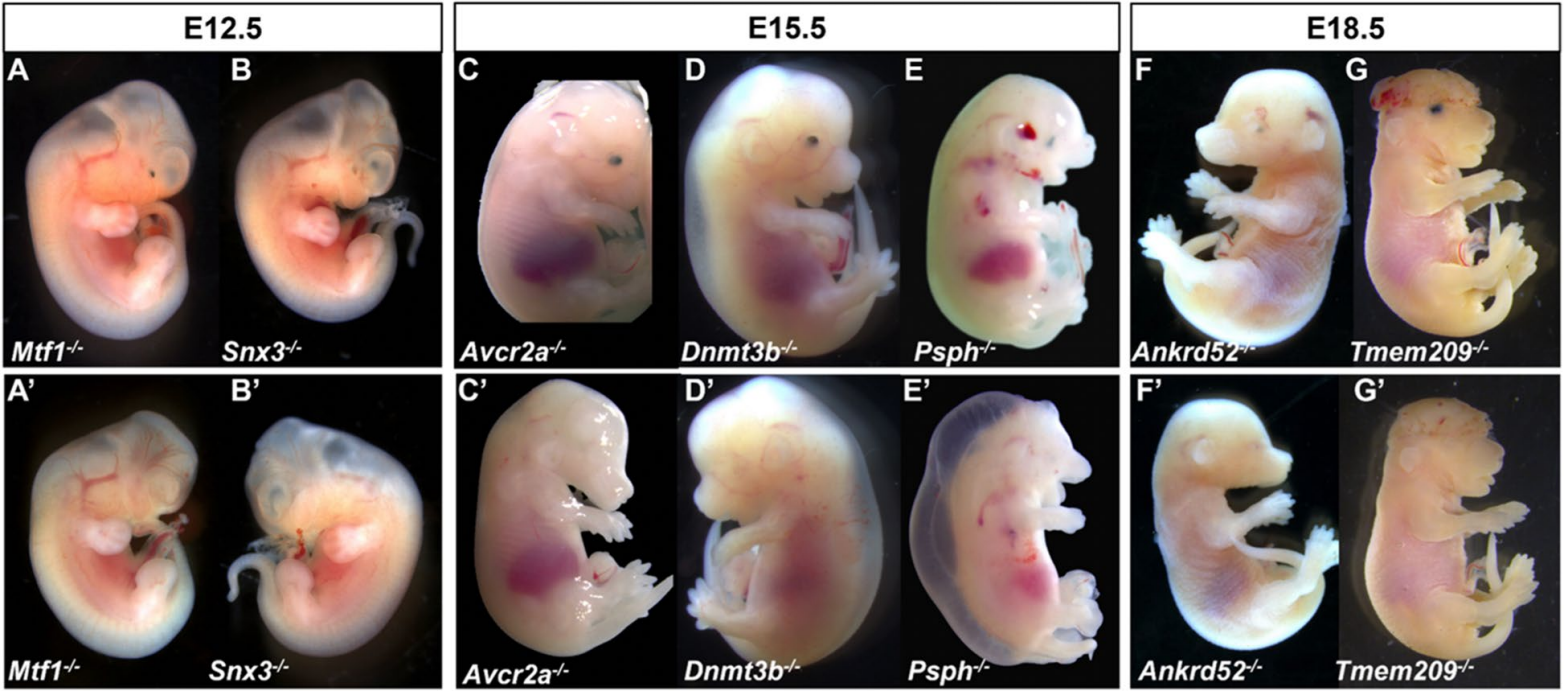
Examples of embryos with homozygous null mutations of genes associated with both microphthalmia (**A**–**G**) and anophthalmia (**A’**–**G’**) at different stages of development. Typically developing mice (WT, **A**–**D**) on C57BL/6N background are shown in Fig. 1. Note the presence of additional anomalies such as short or absent mandible (**C’**, **D**, **D’**, **E**, **E’**, **F**, **F’**), oral cleft (**D**, **E**, **E’**, **G**, **G’**), exencephaly (**G**, **G’**), and moderate to severe edema (**D**, **D’**, **E’**)

A search of the IMPC database for anophthalmia revealed 24 knockout lines with documented evidence of absent eyes in embryos: *Atp13a1*, *Cep135*, *Dzip1l*, *Elavl1*, *Ercc4*, *Faf2*, *Focad*, *Fuz*, *Gabpa*, *Ggnbp2*, *Hesx1*, *Ino80c*, *Lrrc8a*, *Rab34*, *Rbm45*, *Rexo1*, *Slc25a1*, *Slc36a1*, *Snx3*, *Tbc1d32*, *Tctn3*, *Ubn2*, *Uggt1*, and *Zfp503*. Representative examples are shown in Fig. 1. A similar search of DR17 using the term microphthalmia resulted in 22 genes significant for the small eyes phenotype: *Aldh1a3*, *Aff4*, *Bmp4*, *Cdk4*, *Cox6b1*, *Cxcr4*, *Dync1li1*, *Eef1d*, *Fgd1*, *Gne*, *Grh12*, *Mab21l2*, *Maf*, *Med13l*, *Mllt10*, *Mthfd2*, *Pex6*, *Phgdh*, *Ssr1*, *Stim1*, *Tdo2*, and *Vps26c* (Fig. 2). Several genes (*n* = 16) resulted in embryos expressing both microphthalmia and anophthalmia, including *Acvr2a*, *Anapc15*, *Ankrd52*, *Axin2*, *Bmi1*, *Dnmt3b*, *Inpp5e*, *Mmachc*, *Mtf1*, *Pax6, Psph*, *Pygo2*, *Rgl1, Snx3*, *Tmem209*, and *Togaram1* (Fig. 3).

**Fig. 4:**
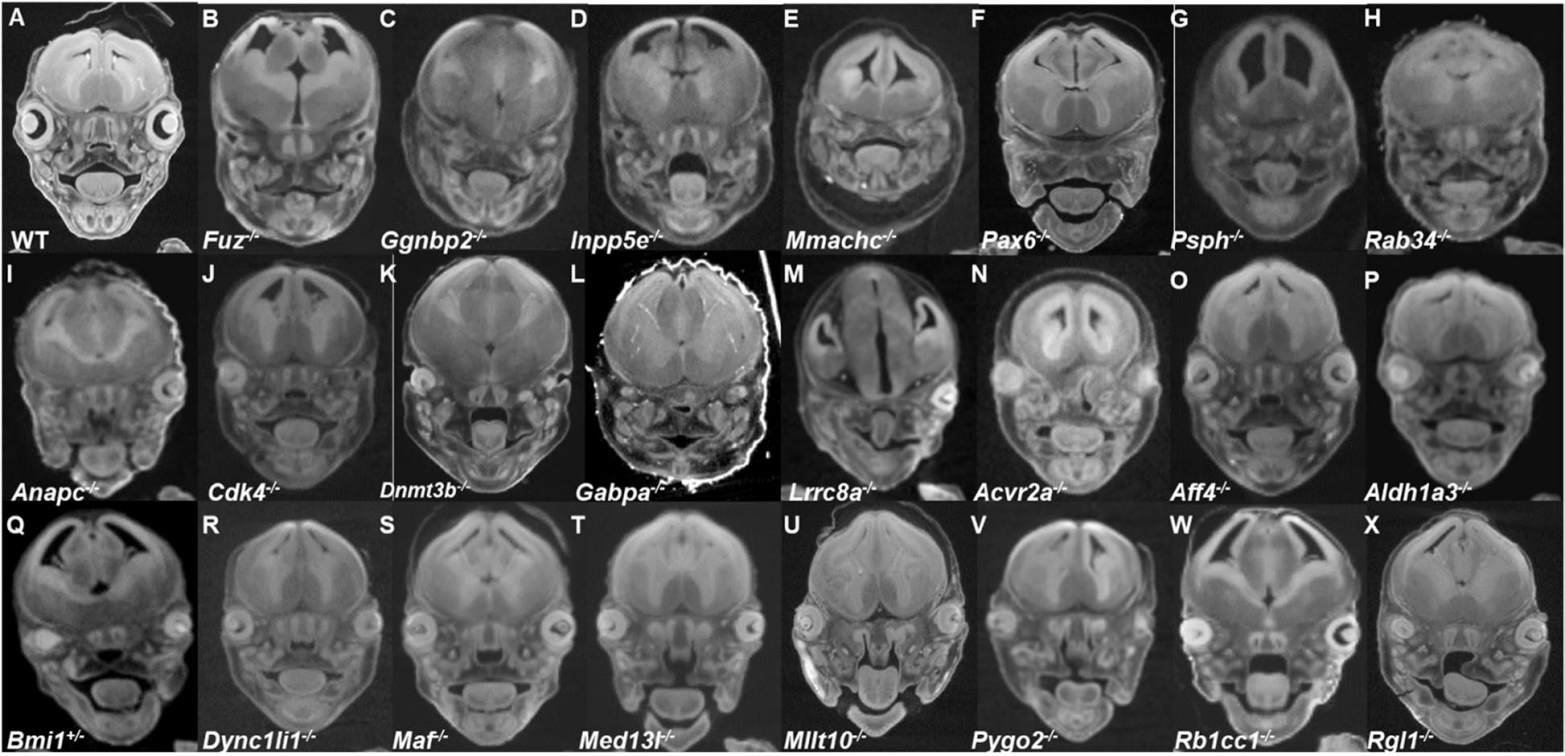
MicroCT (μCT) images of eye abnormalities in E15.5 null (**B**–**P**, **R**–**X**), heterozygous (**Q**), and WT (**A**) embryos. Eye anomalies ranging in severity from bilateral anophthalmia (**B**–**H**), anophthalmia with or without microphthalmia (**I**–**M**), and various degrees of microphthalmia (**N**–**X**) are shown. Additional μCT data are available on the IMPC portal

**Fig. 5:**
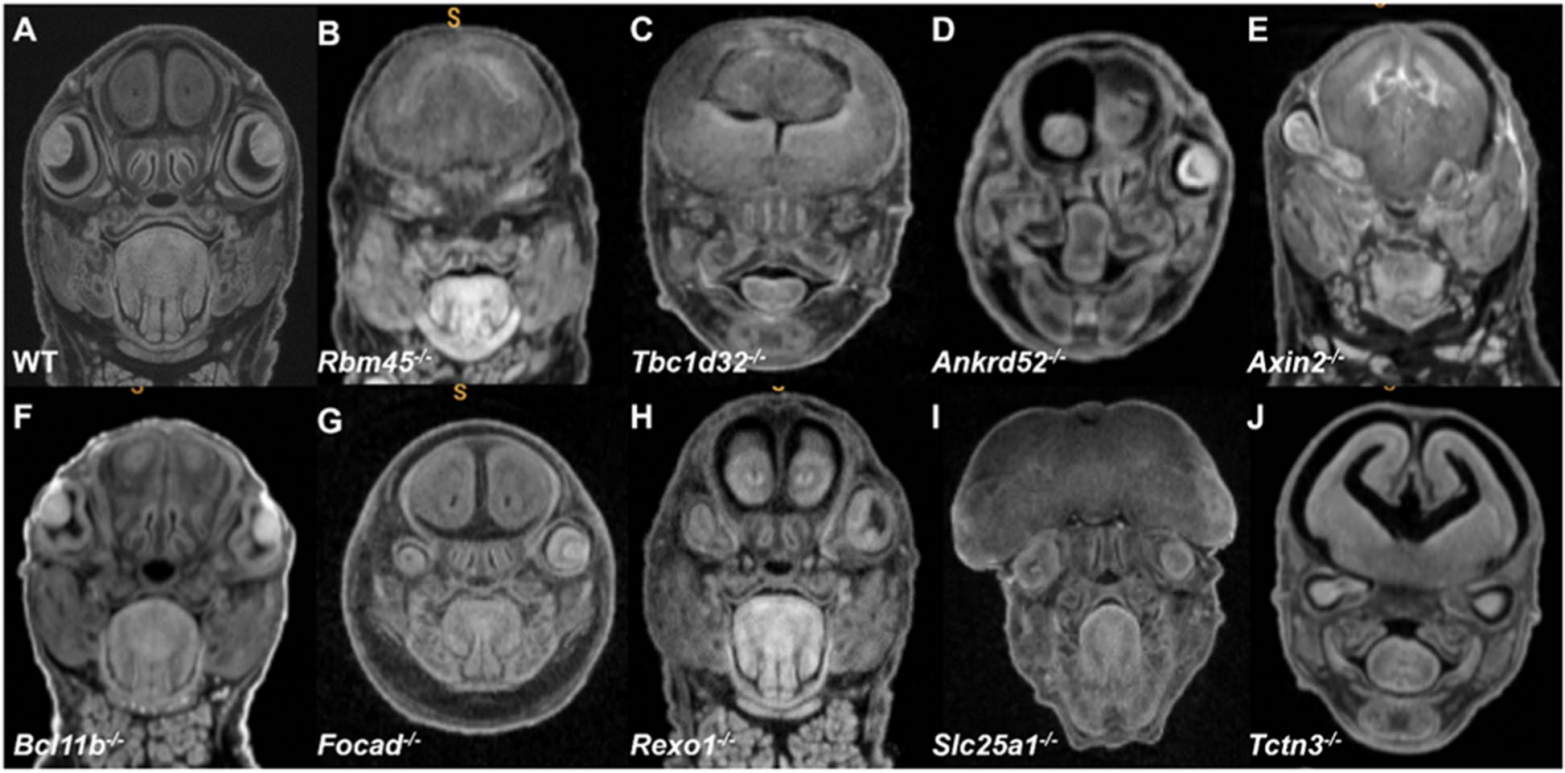
MicroCT images of eye abnormalities in E18.5 null (**B**–**J**) and WT (**A**) embryos. Eye anomalies ranging in severity from bilateral anophthalmia (**B**, **C**), anophthalmia (**D**, **E**), and various degrees of microphthalmia (**F**–**J**) are shown. Additional μCT data available on the IMPC portal

**Fig. 6:**
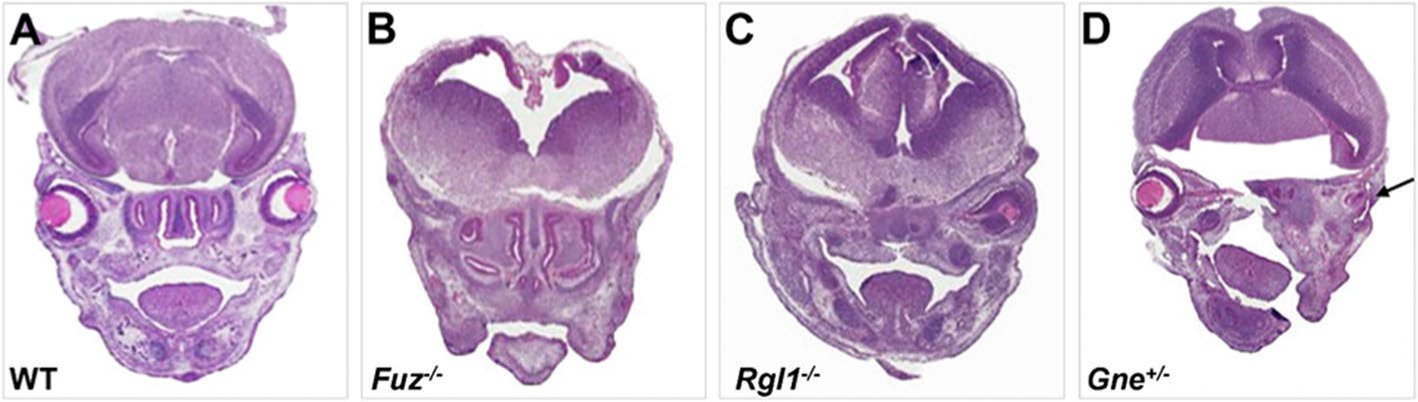
Coronal sections of E15.5 WT (**A**), homozygous (**B**, **C**), and heterozygous (**D**) E15.5 embryos stained with hematoxylin and eosin showing examples of MAC phenotypes. Arrow (**D**) indicates the presence of ocular remnant

Facilities at some IMPC centers were equipped with μCT capability and were thus able to document ophthalmic abnormalities with high resolution in E15.5 and E18.5 mutants and contrast these findings to littermate WT mice (Figs. 4 and 5). These images show that the MAC spectrum ranged in severity from mild microphthalmia with small discernible eyes, severe microphthalmia cases where there is no visible eye, but ocular remnants are found within the orbital sockets, to anophthalmia, with embryos having no visible eyes along with an empty space within the orbit. Histology examination of a subset of these lines at E15.5 confirmed these phenotypes (Fig. 6).

**Fig. 7:**
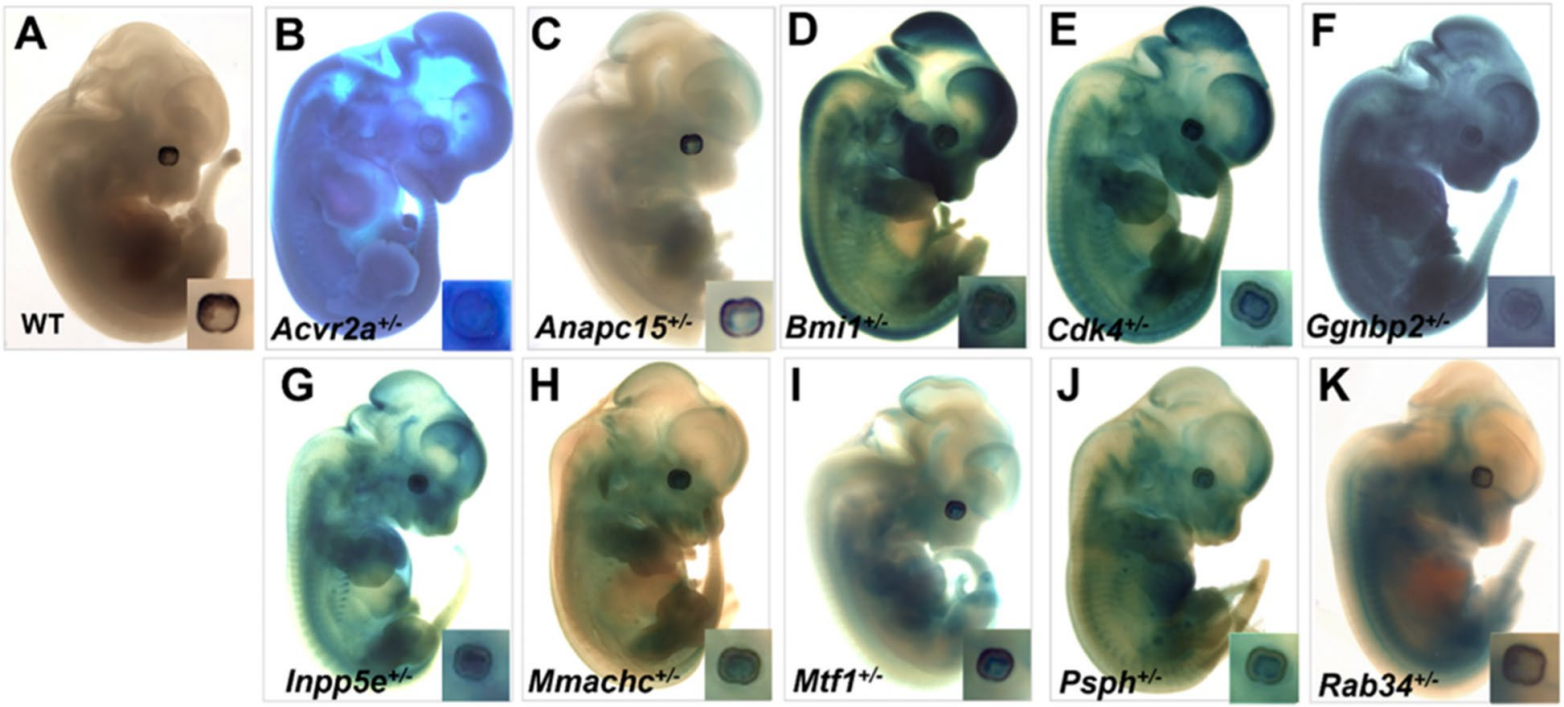
Examples of whole embryo (E12.5) LacZ histochemistry within heterozygous C57BL/6N embryos (**A-K**). Heterozygous embryos were chosen since most appear phenotypically normal. Positive LacZ is taken as a surrogate of endogenous gene expression. Magnification of the eye (inset) shows the positive staining in the ocular tissues in each case

To verify the embryonic expression of the targeted genes in ocular tissues, we examined HET E12.5 embryo staining for β-galactosidase activity, taking advantage of the inserted *Lac*Z reporter cassette in the targeted allele. Heterozygous embryos were chosen rather than HOM in order to approximate a normal gene expression pattern and normal gross embryonic morphology (Fig. 7). We identified positive staining within the eyes in every knockout line for which corresponding LacZ-stained E12.5 embryos were available (Fig. 7B–K), in contrast to LacZ stained WT embryos (Fig. 7A); magnification of the eye (insets) shows positive staining within the ocular tissues. The inset photos are modified to enhance contrast to better visualize the ocular LacZ staining pattern. The positive confirmation of ocular gene expression in the presumably normal eye of all available heterozygous embryos lends credibility to the mechanistic requirement for these genes in early eye development.

**Fig. 8:**
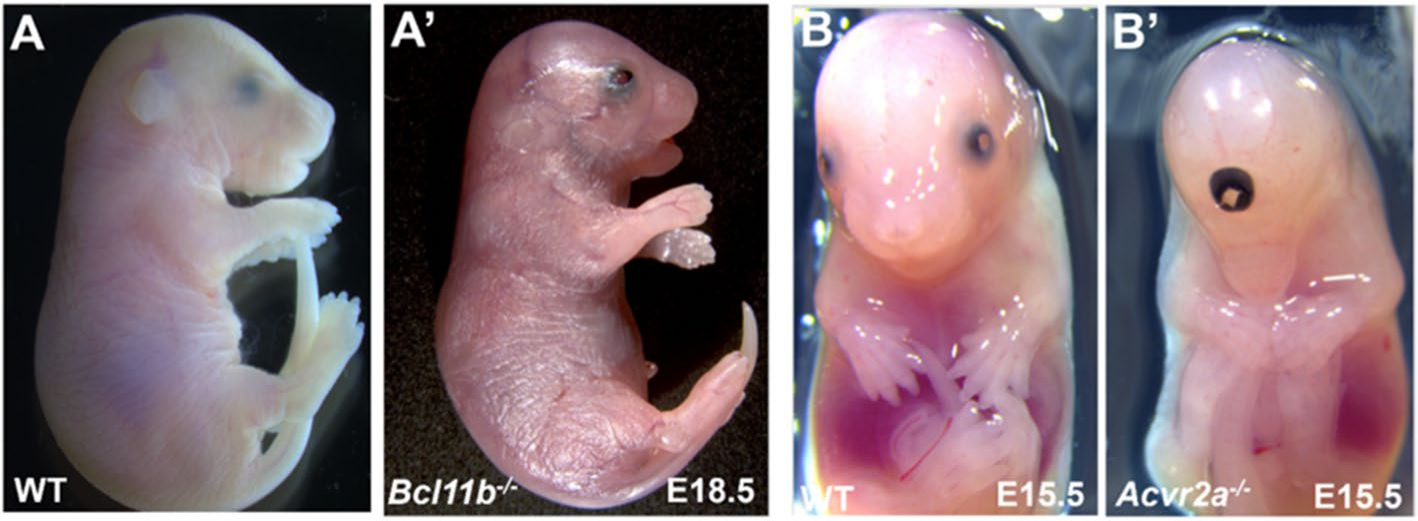
Examples of eye malformations in knockout embryonic mice different from microphthalmia and/or anophthalmia. E18.5 *Bc1llb* null mutant (**A’**) has abnormal eyelid fusion compared to wild-type C57BL/6N (**A**). E15.5 *Acvr2a* null mutant exhibits cyclopia (**B’**) compared to wild-type C57BL/6N mice (**B**)

We performed a literature search to determine the degree to which these 74 genes had established roles in eye development and MAC spectrum diseases. Of the 74 MAC genes identified in mouse embryos among the 8267 single gene mutant lines produced and phenotyped by the IMPC to date, 27 lines had published knockout models in peer reviewed publications, of which 15 reported eye abnormalities (with 9 publications reporting a MAC spectrum phenotype), and 12 KO (knockout) models with no eye phenotypes recorded. Taken together, the 47 unpublished knockout lines and the 12 published knockouts with unreported eye anomalies comprise a total of 59 genes related to early eye formation which were previously unrecognized as being associated with eye development (Additional file 1: Table S2). Of the 15 genes with published eye anomaly reports, the results from the IMPC screen for MAC phenotype confirmed these previously published mouse models with MAC spectrum, such as *Bcl11b*^*−/−*^ with gross morphology findings of abnormal eyelid fusion in E18.5 embryos (Fig. 8A), as reported in the extant KO mouse model [15]; *Pax6*^*−/−*^ and *Inpp5e*^*−/−*^ with anophthalmia [16, 17]; *Axin2*^*−/−*^ with coloboma, microphthalmia, and abnormal eyelid fusion [18]; and *Psph*^*−/−*^ showing strong correlation with the phenotypes (abnormal eye muscle, optic stalk, optic cup, lens morphology, absent lens) reported in a *Psph* KO mouse model cataloged by the Deciphering the Mechanisms of Developmental Disorders (DMDD) program [19].

Nineteen of the 59 genes with unpublished mouse findings had a reported eye phenotype in humans either from existing literature and/or from syndromes indexed in OMIM. As such, our study provided a mouse correlate for a holoprosencephaly-suspected gene from a case report, *ACVR2* [20], with cyclopia (Fig. 8B) observed in *Acvr2a*^*−/−*^ E15.5 embryos confirming the phenotype. Similarly, the microphthalmia phenotype in *Aff4*^*−/−*^ E15.5 embryos is consistent with the higher incidence of eye anomalies and cataracts in patients with CHOPS syndrome many of whom also exhibit heterozygous mutations of the *AFF4* gene [21].

Importantly, these 40 genes (*Acvr2a*, *Alg10b*, *Anapc15*, *Ankrd52*, *Atp13a1*, *Cox6b1*, *Cxcr4*, *Dzip1l*, *Eef1d*, *Ercc4*, *Faf2*, *Focad*, *Gabpa*, *Ggnbp2*, *Gne*, *Ino80c*, *Laptm4b*, *Med1*, *Med13l*, *Mllt10*, *Mtf1*, *Mthfd2*, *Pcnp*, *Phgdh*, *Pigq*, *Rab34*, *Rbm45*, *Rexo1*, *Rgl1*,*Slc25a1*, *Slc36a1*, *Ssr1*, *Stl4*, *Stim1*, *Tmem209*, *Ube2f*, *Ubn2*, *Uggt1*, *Vps26c*, *Zfp503*) represent genes that contribute to early eye development that were previously unrecognized.

In order to identify the novel pathways that may be implicated in early eye formation, we utilized the Panther tool [22] within Gene Ontology [23, 24] to determine which pathways are important for early eye development in the 74 IMPC knockout lines. This tool revealed a number of pathways that are known to be important in early eye development based on the analysis of the 114 gold standard MAC spectrum genes (Additional file 1: Fig. S1). There were several pathways important in early eye formation in our 74 IMPC knockout lines that were not implicated in the established gold standard genes. One of these was the serine-glycine biosynthesis pathway (Additional file 1: Fig. S2). There were also a number of evolutionarily conserved pathways common to both our IMPC knockout lines and the gold standard list including the Hedgehog, WNT, and the TGFβ signaling pathways (Additional file 1: Fig. S3).

We used the STRING biological database software to predict protein-protein interactions within the 74 genes from the IMPC. Among the analysis of these 74 genes, STRING produced a network of predicted interactions between many of the gene products. The 114 genes from the gold standard MAC spectrum disease gene list were analyzed similarly for predicted interaction networks. When comparing the 74 genes generated from the IMPC database and the 114 genes from the gold standard list, eight genes (*ALDH1A3*, *BMP4*, *MAB21L2*, *MAF*, *ME13L*, *PAX6*, *SNX3*, and *TBC1D32*) overlapped between the two groups (Additional file 1: Fig. S4). Cytoscape was used to merge the two networks together to produce a broader picture of interactions between eye development proteins. There were a total of 180 genes within the merged network: 74 genes from IMPC mouse knockout models, 114 human MAC spectrum genes, and eight of which were in both groups. Within these STRING interactions, the serine-glycine biosynthesis pathway and members of ciliogenesis and planar polarity effectors (CPLANE) are encircled; genes associated with the signaling pathways regulating pluripotency of stem cells are identified by stars (Additional file 1: Fig. S5). In order to incorporate both our findings from Gene Ontology regarding the serine-glycine metabolism and signaling pathways regulating pluripotency of stem cells and our findings from STRING regarding the specific genes involved within each pathway, we utilized the Kyoto Encyclopedia of Genes and Genomes (KEGG) database [25] within the Database for Annotation, Visualization, and Integrated Discovery (DAVID) program to map out the pathways (Additional file 1: Figs. S2 and S3) [22, 26].

We used CiliaCarta, a publicly available bioinformatic analysis tool [27], to investigate the proportion of genes that have an established functional role in the primary cilium. There were 12 of the 114 of gold standard genes associated with MAC spectrum disease implicated in ciliary function. A similar analysis of our list of IMPC mouse genes identified eight (*Cep135*, *Dzip1l*, *Fuz*, *Inpp5e*, *Pex6*, *Sufu*, *Tctn3*, and *Togaram1*) of the 74 genes that had ciliary function. These twelve human and eight mouse genes and their roles in ciliopathies are summarized in Additional file 1: Table S3.

## Discussion

The IMPC phenotyping pipeline consists of a standardized set of minimally invasive phenotype tests for early adult mice covering 11 organ systems from 4 to 16 weeks of age. Mice are then sacrificed, and samples are collected for a standardized set of terminal tests that include gross pathology, clinical chemistry, immunophenotyping, and histopathology [28]. Approximately 30% of knockout lines are homozygous lethal at various stages of embryonic development [3]. A standardized embryo phenotyping pipeline is used by IMPC centers to identify the window of lethality, characterize embryo phenotypes using gross morphological examination, and identify and quantify abnormalities using 3D imaging. A subset of lines is also analyzed for gene expression pattern with whole mount β-galactosidase (LacZ) staining. Phenotyping tests are done using standardized protocols published on the IMPC website [29]. Data and images collected during phenotyping are transferred to the IMPC Data Coordination Center (DCC), quality-controlled, and analyzed using OpenStats [30]. After curation by the DCC, the data is uploaded to the IMPC website for the biomedical community to freely access. The IMPC database is dynamic with additional data releases several times each year. For example, at the time we initiated this study, the IMPC Data release 11.0 had generated 6900 unique gene knockout mouse lines and completed phenotyping on 6440 of these lines. Our query of DR11 resulted in 63 genes with MAC phenotypes. However, because it included lines with incomplete phenotyping, an up-to-date query (DR17) resulted in the loss of genes having a significant eye phenotype (*n* = 27) and a discovery of new genes with MAC (*n* = 38) for revised total of 74 genes with MAC phenotypes.

Thanks to the effort of the IMPC to produce and analyze sex balanced cohorts of mutant mice for each gene in the mouse genome, forward genetic approaches can be applied to better understand eye development in mammals. In this study, we identified 74 genes associated with a developmental eye defect of the MAC spectrum, in which 59 of these were previously unrecognized in mice and 40 genes novel to humans, providing new avenues of investigation on the molecular mechanisms regulating eye development. Among the 15 genes with published eye phenotypes, 9 of the lines from the IMPC program had eye phenotypes similar to that of published mouse models (*Axin2*, *Adh1a3*, *Bcl11b*, *Fuz*, *Inpp5e*, *Mab21l2*, *Maf*, *Pax6*, *Pygo2*); the remaining 5 lines resulted in embryonic eye phenotypes (*Acvr2a*, *Bmi1*, *Dnmt3b*, *Dync1li1*, *Hesx1*) more severe than the phenotypes of extant models which consisted of septo-optic dysplasia (Hesx1), abnormal retinal vascularization (*Acvr2a*), and retinal and/or photoreceptor degeneration (*Bmi1*, *Dnmt3b*, *Dync1li1*). There was one exception; B*mp4* HOM embryos exhibited a less severe eye phenotype than expected from published model. The fact that the HOM lines generated by the IMPC were embryonic lethal at E15.5 or E18.5 compared to previous models which survive beyond birth may explain why the IMPC mutants had a more severe eye phenotype, consistent with more severe lethality. Wild-type mice of a similar C57BL/6N background showed a rate of microphthalmia in E15.5 embryos of 8.7% (80% females) and anophthalmia in 0.7% (88% females). The sexual dimorphism in this background rate of microphthalmia/anophthalmia warrants further study. Although the rd8 mutation present in C57BL/6N mice may contribute to the MAC phenotype in these 74 lines, the fact that the eye defects were observed in HOM/HEMI embryos (34 of 50 genes) but not in the HET and WT embryos, that many of the lines (*Axin2*, *Bmi1*, *Fgd1*, *Ggnbp2*, *Maf*, *Med13L*,*Pax6*, *Rgl1*,*Top2b*), showed a gene dosage effect with increasing severity or expression with increasing mutated alleles, and that all eye phenotypes are significantly different from WT of the same sex and background point to a direct association between these genes and the MAC spectrum. Analyses of these 74 genes revealed the importance of the serine-glycine pathway, the ciliogenesis and planar polarity effectors (CPLANE) complex, and the signaling pathways for progenitor cell maintenance to be three critical mechanisms required for eye formation.

### Serine-glycine pathway within the folate cycle

Merging the 74 IMPC genes and the 114 MAC spectrum genes using STRING revealed a cluster of interrelated genes that are involved in serine-glycine biosynthesis within the folate cycle. While folate deficiency is associated with eye defects, and components of the serine-glycine pathway have been known to cause congenital eye disease [31–34], the serine-glycine pathway has never previously been associated with the MAC spectrum disorder. Our query also uncovered three novel genes (*Mthfd2*, *Phgdh*, *Slc25a1*) not previously recognized as an underlying fundamental mechanistic factor in MAC spectrum disease and associated with this pathway (Additional file 1: Fig. S2). The serine glycine biosynthesis pathway is important in converting glucose into serine and glycine to meet the cellular requirements for serine [35]. Glucose is imported into cells and then converted into an intermediate, phosphohydroxy pyruvate, through catalysis by phosphoglycerate dehydrogenase (PHGDH). This intermediate is then converted into P-serine by phosphoserine aminotransferase (PSAT1), then converted into serine by phosphoserine phosphatase (PSPH) and further into glycine by serine hydroxymethyl transferase (SHMT) [36]. Mutations PHGDH, PSPH, and PSAT1 in the serine-glycine pathway have been associated with Neu-Laxova syndrome, which is characterized by a spectrum of phenotypes that vary in expression but usually manifests by perinatal lethality, microcephaly, skeletal, and skin anomalies [37]. Eye and brain defects including shortened or absent eyelids, proptosis, microphthalmia, microcephaly, lissencephaly, and agenesis of the corpus callosum, and neural tube defects have been reported [38, 39].

We hypothesize that mutations of genes within the serine-glycine pathway contribute to defects in eye development, as glycine and serine are important sources of 1C units to the folate one-carbon metabolism (FOCM) via the cleavage of glycine by glycine decarboxylase (GLDC) [40]. Interestingly, *Gldc* null embryos exhibited high incidence of neural tube defects (57%) and microphthalmia or anophthalmia (30%) [40]. Moreover, serine and serine-derived metabolites such as sphingolipids have been shown to support critical functions in maintaining retina integrity [41]. We postulate that by having defects within serine-glycine biosynthesis, there will be decreased conversion of serine to glycine, which leads to decreased transfer of one-carbon units to the mitochondrial FOCM. This in turn can lead to neural tube and subsequent (secondary) embryonic eye defects on the MAC spectrum during embryogenesis.

### Ciliogenesis and planar polarity effector (CPLANE) complex

Our IMPC query for MAC phenotype-associated genes included several members of the CPLANE complex including *Gabpa* and *Dzip1l*, which were not previously associated with eye defects. Primary cilia are important components of the retinal photoreceptors, the sensory layer of the retina. The outer segment of the photoreceptors is a highly modified primary cilium which has evolved to play a crucial role in vision [42]. Changes in primary cilium morphology or structure have been associated with retinal diseases such as macular diseases, retinitis pigmentosa, Leber congenital amaurosis, cone dystrophies, and other photoreceptor diseases [43, 44]. In addition to the CPLANE genes, our search uncovered a number of additional ciliopathy genes associated with multi-systemic, brain, kidney, and eye ciliopathies (*Cep135*, *Innp5e*, *Pex6*, *Togaram1*, see Additional file 1: Table S3). The members of the CPLANE complex, by contributing to the formation of primary cilia, are important regulators of the hedgehog and WNT signaling pathways [45]. Restricted spatial distribution of SHH is essential for the specification of eye progenitor cells [46], as SHH-mediated induction of embryonic tissues including eye formation is dependent on the establishment of SHH morphogenic gradients [45]. The association between aberrant primary cilium formation, SHH expression, and eye defects have been demonstrated for SUFU, Fuz, JBTS17/*Cplane1*, TBCD132, and TCTN3. In addition, while Gabpa and Dzip1l have been previously associated with ectopic SHH expression [47, 48], our data now confirmed a role for these two genes in cilia-SHH interactions and to the pathogenic mechanisms underlying eye defects. Specific genes of the CPLANE complex can affect the planar cell polarity (PCP) cascade with ensuing ciliopathy-mediated defects resulting in severe disruption of organogenesis during development including the eyes. Examination of μCT images of E15.5 HOM *Fuz* mutant embryos uncovered the extent of embryonic defects resulting from impaired primary cilia formation, and likely subsequent impaired sonic hedgehog (SHH) signaling, and planar cell polarity (PCP) regulation; these include absent lungs and pituitary, agenesis of the tongue, hypoplastic mandible, cleft palate, Meckel’s cartilage malformation, dextrocardia and heart anomalies, absent or polycystic kidneys, and neural tube defects (data not shown).

### Signaling pathways regulating pluripotency of stem cells

STRING analysis also revealed a cluster of genes that are involved in three critical signaling pathways that regulate stem cell differentiation and support critical processes (cell fate determination, patterning, and polarity) during embryogenesis, including novel genes. Studies show that early eye specification, optic cup differentiation, and eye morphogenesis are regulated by the integrated cross-talk between these signaling pathways and transcription factors [49–52]. Knockouts of genes involved in the SHH, the transforming growth factor beta (TGFβ)/bone morphogenetic proteins (BMP), and the Wingless-related integration site (WNT) signaling pathways have been associated with embryonic eye defects [53–55]. Unexpectedly, our query of the IMPC database for embryonic eye defects revealed genes associated with the TGFβ (*Bmp4* and *Acvr2a*), WNT (*Axin2* and *Pygo2*), and SHH (*Fux*, *Sufu*, *Gabpa*, *Cplane1*, *Tctn2*, and *Tbc1d32*) signaling pathways and importantly included novel genes (*Avcr2a* and *Gabpa*). Additional work is required to uncover the precise role of these novel genes in these critical embryonic signaling pathways.

#### ACVR2A and its relationship to SHH and cyclopia

Cyclopia occurs due to optic vesicle fusion, creating a singular eye and causing further neural abnormalities that are incompatible with life [10]. This is the most extreme manifestation of holoprosencephaly (HPE), a condition that results from abnormal forebrain formation and closure during early embryonic development [56]. The etiology of HPE is complex and includes genetic mutations (several key loci have been identified, [56]) and teratogenic insults such as exposure to the naturally occurring alkaloid cyclopamine [57], retinoic acid [58], or cholesterol deficiency [59]. These teratogens were shown to abrogate the hedgehog signaling pathway [60], specifically SHH, which is an important morphogen that regulates CNS patterning and eye morphogenesis [61]. SHH signaling within the neuroepithelium modulates the formation of the optic cup, optic stalk, and the retinal pigmented epithelium alongside the dorso-ventral axes. *Shh* knockout mice show embryonic lethality by E9.5, with a phenotype showing cyclopia [62, 63]. Activin receptor type 2A (ACVR2A) is a transmembrane receptor that binds to activin A. *Acvr2a* is largely expressed within the head of zebrafish and plays a role in anterior-posterior patterning. When depleting *acvr2a* in zebrafish, these lineages exhibited ocular phenotypes such as swollen eyes that were mediolaterally displaced suggesting a role in eye field patterning [64]. In humans, one study showed that one patient out of a cohort of 136 individuals with signs of HPE had a deletion within ACVR2A, which led them to consider it an “HPE candidate” gene [20]. Within our STRING analysis (Additional file 1: Fig. S5), SHH is within the same network as ACVR2A, which signifies that the sonic hedgehog and activin pathways may interact, but the relationship between ACVR2A, hedgehog signaling and midline defects such as cyclopia warrant further study. A hypothesized model of cross-talk between the SHH, TGFβ, and WNT pathways involving *Acvr2a* is illustrated in Additional file 1: Fig. S3.

## Conclusion

Overall, phenotype screening of embryonic eyes in single gene knockout mouse lines have revealed 40 novel genes that are associated with abnormalities in early eye development. LacZ expression patterns within the heterozygous murine embryos further support these findings by showing gene expression within the eye. Many of these genes are linked to a number of conserved signaling pathways (SHH, WNT, TGFβ), CPLANE complex, as well as to the serine and folate metabolic pathways, which influence cellular proliferation (nucleotide synthesis) and methylation reactions (epigenetics). All of the above are likely to influence multiple cellular functions and morphogenic processes during embryonic development*.* Consequently, the majority of mutant embryos exhibited morphogenic anomalies in addition to the MAC spectrum phenotypes (Additional file 1: Table S1); brain malformations (exencephaly and HPE) were often associated with anophthalmia and craniofacial defects were often observed in mutants with microphthalmia. Similarly, as described for Fuz mutants, embryos with mutation of CPLANE genes exhibited a pleiotropy of defects. In one unique case, a gene (*Acvr2a*) was associated with cyclopia in addition to MAC anomalies confirming human findings in holoprosencephaly cases. This generates hypotheses about possible cross-talk between TGFβ and hedgehog signaling pathways in splitting the embryonic eye field at a very early stage of development. These findings highlight the requirement of SHH, WNT, and TGFβ signaling pathways regulating the pluripotency of stem cells for ocular morphogenesis. Similarly, we confirmed the importance of ciliopathy genes and the CPLANE complex in eye development. Additionally, there a number of genes for which our bioinformatics analysis did not reveal protein interactions or networks such as *Alg10b*, *Atp13a1*, *Eef1d*, *Errc4*, *Focad*, *Laptm4b*, *Rexo1*, *Rgl1*, *Ubn2*, and *Vps26c*. Their precise contribution to early mammalian eye development remains to be uncovered. Gene Ontology and analysis of protein-protein interactions between the mouse genes in this study and established human MAC genes revealed the contribution of previously unrecognized processes such as the serine-glycine biosynthesis pathway for eye development. In individuals with deficiencies of enzymes involved in serine-glycine biosynthesis, these mouse models provide an opportunity to investigate potential therapeutic avenues such as folate, formate, and/or B6 supplementation to reduce the occurrence of eye maldevelopment.

This study has several limitations. The mouse lines presented here in some cases coincide with extraocular developmental anomalies such as exencephaly and craniofacial defects, suggesting they may be examples of secondary anophthalmia. All mice were generated on the C57BL/6N background which carries the *rd8* mutation in *Crb1* and can have several ocular consequences [65–67]. Therefore, it is possible that some of the observed phenotypes are digenic phenomena involving the targeted deleted gene and *Crb1*. The candidate MAC genes in this report require further validation to confirm clinical relevance. In spite of the limitations of this approach, forward genetic screening of IMPC knockout mice is a useful strategy to understand the genetic mechanisms of mammalian eye development, advance leading edge therapeutics, and provide disease models in which to test them. Single gene knockout mouse technology has the capability to improve our understanding of human MAC spectrum disease, an important cause of childhood blindness. We encourage physicians to validate the candidate genes discovered in IMPC mouse mutants in unsolved cases of MAC spectrum disease.

## Methods

### Bioinformatics

We queried the IMPC database (data release 17, 8267 KO mouse lines) for phenotypes associated with significant eye defects in E9.5, E12.5, E15.5, and E18.5 mouse embryos using mammalian phenotype annotation terms (microphthalmia, anophthalmia, coloboma, abnormal eye morphology, abnormal optic vesicle). A significant eye phenotype was defined as 2 or more homozygous embryos or 4 or more heterozygous embryos exhibiting an eye defect. Lines with significant phenotypes were analyzed for statistical significance using the OpenStats tool kit, available as an R package [68]. Mutant genotypes with a *P*-value less than 0.0001 were considered a significant difference from controls at the IMPC level; we identified 57 genes that met this statistical threshold. Additionally, we included lines with a genotype effect with *P*-values greater than 0.0001 and less than 0.05; there were 17 genes that met these criteria. In total, we identified 74 genes with embryo mutants having one or more significant eye phenotype, the vast majority being microphthalmia and anophthalmia.

Using the search engines www.pubmed.gov and www.google.com/scholar, the set of 74 genes was queried within existing literature to identify previously published mouse knockout models with eye abnormalities. To determine if each gene had been associated with eye disease in humans, search terms related to the eyes and its associated structures, such as eye, anophthalmia, microphthalmia, coloboma, or retina were used. For a given gene, if there was not an extant knockout model, or the published knockout reported no ocular phenotype in mouse embryos, and no ocular phenotypes associated with it in humans, it was designated as a “novel MAC gene.” The final literature search looked for review articles and case reports that documented genes in humans related to MAC spectrum disorder, so that a gold standard list of published human genes (*n* = 114) could be created for comparison to the 74 IMPC mouse genes. After converting the 74 mouse gene symbols to the human orthologues, these two groups of genes were analyzed using the STRING [69] biological database software to predict protein-protein interactions within and between the groups, and molecular functions were generated using the Panther tool [22] within Gene Ontology [22, 24, 69]. The relationships between our genes within STRING are characterized with a medium level of confidence (0.40–0.69), high confidence (0.70–0.89), or the highest confidence (0.90–1.0), with most gene relationships falling within the medium level of confidence [70].

### Animals

All institutions involved in this study operated under the regulation and accreditation from their national animal welfare committee [Institutional Animal Care and Use Committee (IACUC) or Animal Care Committee (ACC)]. This ensured that each institution would operate under standardized, ethical procedures that minimized pain and suffering for all mice included in this work. The IMPC utilizes a systematic approach towards phenotyping mice that are either homozygous (HOM) for knockout of a single-gene or heterozygous (HET) for the gene if the homozygous mice are subviable or lethal. For X-linked genes, the knockout male mice are designated hemizygous (HEMI). The strategies utilized for gene knockout can be accessed on http://www.mousephenotype.org/about-ikmc/targeting-strategies.

### Embryo phenotyping

Embryos (E9.5, E12.5, E15.5, and E18.5) were recovered from time-mated heterozygous crosses. Fertilization (0 h) was set as the midpoint of the dark cycle prior to the detection the copulation plugs. Embryos were dissected free from their placenta and membranes in phosphate-buffered saline (PBS minus Ca^++^/Mg^++^), and the yolk sacs were collected for genotyping. Eye development and phenotype were assessed through gross morphological examination of individual embryos under a phase microscope. Eye anomalies were noted using stage appropriate gross morphology annotation terms and photo-documented when possible. For the eye morphology assessment, the examiner was masked to the genotype of the embryos since PCR-based genotyping of the corresponding yolk sacs took place subsequent to the embryo phenotyping process. E15.5 and E18.5 embryos were transferred to warm heparinized (1 U/ml) PBS and euthanized by exsanguination. Individual embryos were submerged in fixative (4% paraformaldehyde/PBS) and rocked at 4 °C overnight or up to 7 days depending on the age/size of the embryo and then stored at 4 °C in PBS containing 0.02% sodium azide until processed for μCT or histology. A minimum of 28 live embryos were collected at each time point including at least eight homozygotes for each knockout mouse line.

### LacZ staining

E12.5 mouse embryos were stained for β-galactosidase activity using X-gal [71]. Embryos were dissected free of extraembryonic membranes in warm PBS, transferred to 12-well plates, and fixed individually in a 10% buffered formalin solution containing glutaraldehyde (8%), EGTA (0.5M), and IGEPAL (10%) for 60 min at room temperature with gentle rocking. The yolk sacs were collected for genotyping at the time of dissection. Following several washes to remove the fixative, the embryos were immersed in the X-gal solution and stained for ~ 18 h at room temperature, protected from light with moderate agitation. Embryos were postfixed in 10% buffered formalin, cleared with sequential solutions of glycerol and potassium hydroxide (1%) and stored in 70% glycerol until image acquisition and evaluation of staining pattern.

### Micro-computed tomography (μCT) imaging

For μCT, individual E15.5 and E18.5 embryos were incubated in a hydrogel stabilizing solution (4% PFA, 4% acrylamide, 0.05% bis-acrylamide, 0.25% VA044 Initiator, 0.05% saponin in PBS) for 3 days at 4 °C to preserve tissue integrity. Thereafter, the vials containing the embryos were placed in a desiccation chamber and saturated with nitrogen gas to replace the air and embryos were incubated in a 37 °C water bath. Finally, embryos were removed from the encasing hydrogel, swiped clean, and immersed in a Lugol solution [0.7% iodine solution (0.1N)] for at least 24 h at room temperature while rocking and then embedded in 1% agarose and oriented for μCT imaging. Embryos were imaged using a high-resolution MicroXCT-200 specimen CT scanner (Carl Zeiss X-ray Microscopy). The CT scanner has a variable X-ray source capable of a voltage range of 20–90 kV with 1–8 W of power. Embryos were placed on the scanner’s sample stage, which has a submicron level of position adjustments. Scan parameters were adjusted for each embryonic age, based on the manufacturers recommended guidelines. A total of 1600 image projections were obtained over a 360° rotation. The camera pixels were binned by two to increase signal to noise in the image and the source-detector configuration resulted in a voxel size of 11.4791 μm. Images were reconstructed with a smoothing factor of 0.7 and a beam hardening of 0.2 into 16-bit values with common global minimum and maximum values for proper histogram matching.

### Histology

Embryos were dehydrated in a graded series of alcohol washes, cleared in xylene, embedded in paraffin, and cut in serial frontal sections (5 μm) using a microtome (Thermo Scientific Micron HM355S, Leica Biosystems, Deer Park IL) and collected on slides for hematoxylin and eosin staining using a standard protocol. Slides were scanned using a ScanScope XT Slide Scanner and viewed with the Image Scope software, version 10.2.2.2319 (Aperio).

## Supplementary Information

**Additional file 1: Table S1.** Knockout mice exhibiting phenotypic embryonic eye defects. The embryonic eye defects were divided into four categories: Abnormal eyelid fusion, abnormal optic vesicle formation, microphthalmia, and anophthalmia. If a knockout strain of the gene has already been created, a PMID is listed, along with a description of the ocular defect, age, and zygosity of the mice upon diagnosis if pertinent. If there is an ocular defect present within humans (Human Eye PMID), or if there is a weak, potential correlation between the gene and an ocular defect in any species besides mice and humans (Associated Phenotypes), the PMID is listed as well. Any systemic co-phenotypes (numerical code listed below) associated with either homozygous or heterozygous mice are listed, along with the description of the heterozygous eye phenotype. From the IMPC database, if a photo of ocular defects was present in heterozygous adult mice, the type of photo taken was described in the spreadsheet. Additionally, from the same database, the phenotypic embryonic eye defects were labelled for each knockout strain, along with the age the mice were diagnosed with the defect (E9.5, E12.5, E15.5, E18.5), their homozygous viability, the zygosity of the mice with the phenotypic eye defect, and a ratio of mutant KO to normal KO mice by sex. In conclusion, the IMPC yielded 74 genes that created knockout strains with an embryonic eye defect, 27 of which had already had previously published mouse knockouts, but only15 of these knockouts exhibiting an eye abnormality. There are a total of 59 genes that are associated with embryonic eye defects in mice of which 19 genes had a previously reported human eye phenotype. Therefore, there are a total of 40 genes not previously associated with eye defects; these are bolded and highlighted in red in the first column of the table. 1 = Endocrine/exocrine 11 = Respiratory 2 = Taste/olfaction 12 = Cardiovascular 3 = Ear, hearing, vestibular 13 = Behavior, Neurologic 4 = Craniofacial 14 = Metabolism/homeostasis 5 = Muscle phenotype 15 = Growth and Body Size 6 = Immune/Hematopoietic 16 = Reproductive System 7 = Skeletal Phenotype 17 = Embryonic 8 = Limbs, digits, tail 18 = Mortality, aging 9 = Integumentary/Pigmentation 19 = Eyes 10 = Digestive 20 = Urinary. **Table S2.** The Gold standard list of 114 genes was created from a list of previously published genes that contribute to MAC spectrum disorders in humans. The 74 IMPC list of genes is listed beside it, and the genes present on both the gold standard list and IMPC list are highlighted in yellow. References from which the gold standard list was curated from are listed on the column to the furthest right (PMID or DOI). **Fig. S1.** Pie graphs demonstrating and comparing the molecular pathways of both the 74 IMPC genes and the 114 gold standard genes, using the Panther function on Gene Ontology. Pathways with the red box are only implicated from the mouse data from our genes. The genes implicated within the pathways with the red box are labeled next to the pathway. **Fig. S2.** Serine-Glycine biosynthesis pathway derived from the KEGG pathway on DAVID, based on pathways and gene interactions predicted by gene ontology and STRING analysis. IMPC genes with MAC phenotypes (blue) are highlighted. **Fig. S3.** Interrelationships between pathways involved in stem cell maintenance and proliferation derived from the KEGG pathway on DAVID. Established MAC genes (pink) and IMPC genes with MAC phenotypes (blue) are highlighted. Red indicates a gene in both groups, and SOX2 is highlighted (pink) as an established critical MAC spectrum disease gene. **Fig. S4.** Analysis of protein-protein interactions between the IMPC genes and the Gold Standard genes within a merged network. Cytoscape was the software platform used to merge the two networks together, and the protein interactions between the two groups of genes were analyzed using the STRING biological database software program. The arrows point towards genes that are initially not incorporated into STRING clusters until merged together within the Cytoscape platform. **Fig. S5.** Analysis of protein-protein interactions in the 74 IMPC genes (left) and the 114 Gold Standard genes (pink, right) using STRING biological database software. Among the IMPC genes, strongly significant (*P*<0.0001, green) and conventionally significant (*P*<0.05, yellow) genes are shown. Red genes are those that also appear on the list of gold standard MAC genes. Genes with stars (*n*=6) are members of signaling pathways regulating pluripotency of stem cells. Clusters encircled represent the serine-glycine pathway (red box) and Cplane1 complex (blue box). **Table S3.** Genes implicated in ciliopathies from both the IMPC and Gold Standard list of genes. The genes on column A represent IMPC genes that are implicated in ciliopathies, while the genes in column F represent Gold Standard genes that are implicated in ciliopathies.

## Data Availability

All data generated or analyzed during this study are included in this published article and its supplementary information files. Data described in the manuscript may be made available upon request pending approval from all authors: Data is available on the International Mouse Phenotyping Consortium (IMPC) portal: https://wwww.mousephenotype.org/.

## Abbreviations

ACC: Animal Care Committee
BMP: Bone morphogenetic proteins
CPLANE: Ciliogenesis and planar polarity effectors
DAVID: Database for Annotation, Visualization and Integrated Discovery
DCC: Data Coordination Center
DMDD: Deciphering the Mechanisms of Developmental Disorders
FOCM: Folate one-carbon metabolism
GLDC: Glycine decarboxylase
HEMI: Hemizygous
HET: Heterozygous
HOM: Homozygous
HPE: Holoprosencephaly
IACUC: Institutional Animal Care and Use Committee
IMPC: International Mouse Phenotyping Consortium
KEGG: Kyoto Encyclopedia of Genes and Genomes
KO: Knockout
MAC: Microphthalmia, anophthalmia, and coloboma
PBS: Phosphate-buffered saline
PCP: Planar cell polarity
PHGDH: Phosphoglycerate dehydrogenase
PSAT1: Phosphoserine aminotransferase
PSPH: Phosphoserine phosphatase
SHMT: Serine hydroxymethyl transferase
TGFβ: Transforming growth factor beta
WNT: Wingless-related integration site
WT: Wild-type
μCT: Micro-computed tomography
