# Supplementary material for: Genome-wide screening reveals the genetic basis of mammalian embryonic eye development
Source: BMC Biol. 2023 Feb 3;21:22. doi: 10.1186/s12915-022-01475-0 (PMC9898963; doi:10.1186/s12915-022-01475-0)
Supplement: Supplementary file 1 — Additional file 1: Table S1. Knockout mice exhibiting phenotypic embryonic eye defects. The embryonic eye defects were divided into four categories: Abnormal eyelid fusion, abnormal optic vesicle formation, microphthalmia, and anophthalmia. If a knockout strain of the gene has already been created, a PMID is listed, along with a description of the ocular defect, age, and zygosity of the mice upon diagnosis if pertinent. If there is an ocular defect present within humans (Human Eye PMID), or if there is a weak, potential correlation between the gene and an ocular defect in any species besides mice and humans (Associated Phenotypes), the PMID is listed as well. Any systemic co-phenotypes (numerical code listed below) associated with either homozygous or heterozygous mice are listed, along with the description of the heterozygous eye phenotype. From the IMPC database, if a photo of ocular defects was present in heterozygous adult mice, the type of photo taken was described in the spreadsheet. Additionally, from the same database, the phenotypic embryonic eye defects were labelled for each knockout strain, along with the age the mice were diagnosed with the defect (E9.5, E12.5, E15.5, E18.5), their homozygous viability, the zygosity of the mice with the phenotypic eye defect, and a ratio of mutant KO to normal KO mice by sex. In conclusion, the IMPC yielded 74 genes that created knockout strains with an embryonic eye defect, 27 of which had already had previously published mouse knockouts, but only15 of these knockouts exhibiting an eye abnormality. There are a total of 59 genes that are associated with embryonic eye defects in mice of which 19 genes had a previously reported human eye phenotype. Therefore, there are a total of 40 genes not previously associated with eye defects; these are bolded and highlighted in red in the first column of the table. 1 = Endocrine/exocrine 11 = Respiratory 2 = Taste/olfaction 12 = Cardiovascular 3 = Ear, hearing, vestibular 13 = Behav [file 12915_2022_1475_MOESM1_ESM.zip › Supplemental Figures Combined- FINAL FINAL-4.docx]

**Supplementary Information for GENOME-WIDE SCREENING OF KNOCKOUT MICE**

**REVEALS GENES INVOLVED IN EMBRYONIC EYE DEVELOPMENT**

Justine M. Chee^1^, Louise Lanoue^2^, Dave Clary^2^, Kendall Higgins^3^, Lynette Bower^2^, Ann Flenniken^4,5^, Ruolin Guo^4,19^ David J. Adams^6^, Fatima Bosch^7^, Robert E. Braun^8^, Steve DM. Brown^9^, H-J Genie Chin^10^, Mary E. Dickinson^11^, Chi Wei Hsu^11^, Michael Dobbie^12^, Xiang Gao^13^, Sanjeev Galande^14^, Anne Grobler^15^, Jason D. Heaney^11^, Yann Herault^16^, Martin Hrabe de Angelis^17^, Fabio Mammano^18^, Lauryl MJ. Nutter^4,19^, Helen Parkinson^20^, Chuan Qin^21^, Toshi Shiroishi^22^, Radislav Sedlacek^23^, J-K Seong^24^, Ying Xu^25^, The International Mouse Phenotyping Consortium^§^, Brian Brooks^26^, Colin McKerlie^19,27^, K. C. Kent Lloyd^2,28^, Henrik Westerberg^9^, Ala Moshiri^29*^

Corresponding author: Ala Moshiri MD, PhD

Email: amoshiri@ucdavis.edu

**This PDF file includes:**

Supplementary text

Figures S1 to S5

Tables S1 to S3

SI References

**Supplemental Figures**

**
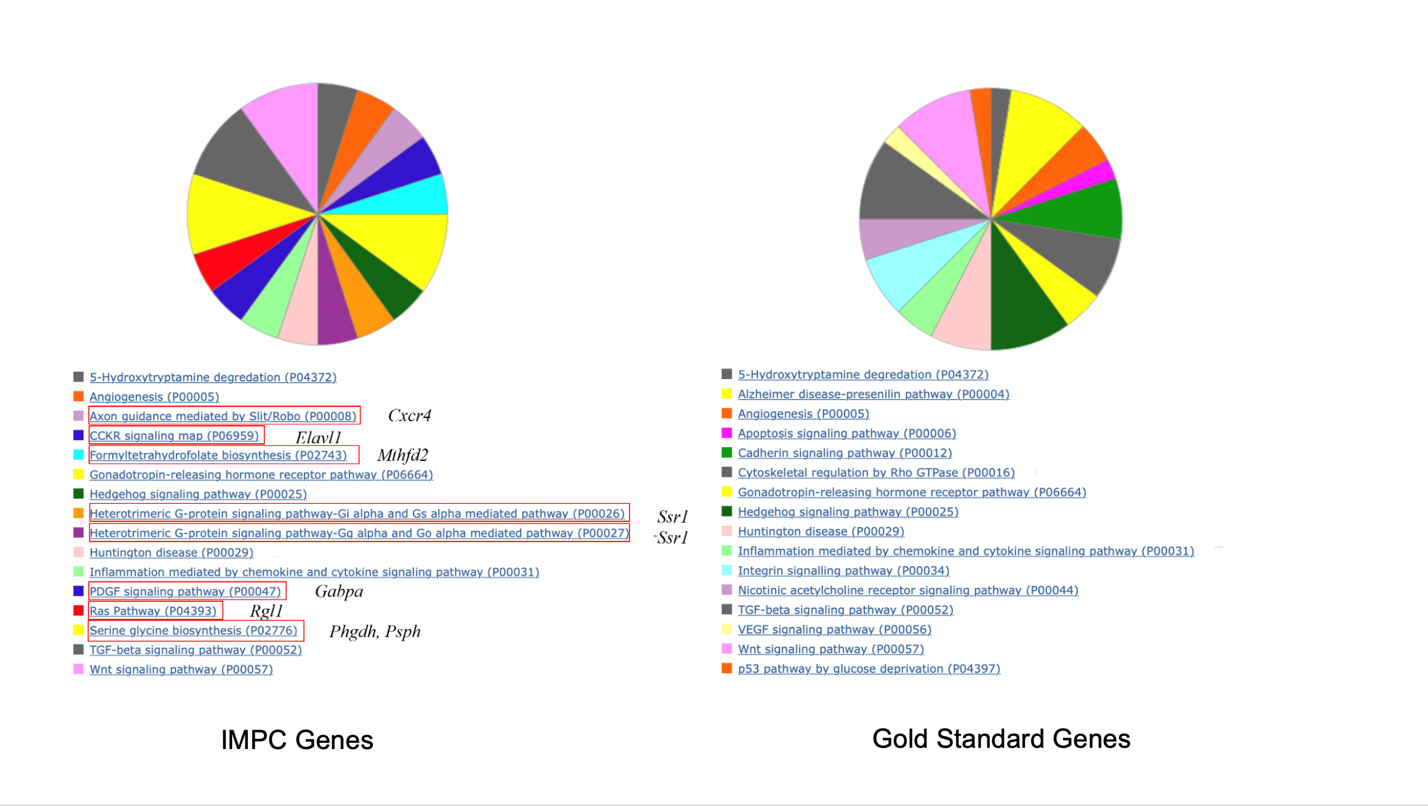
**

**Supplemental Figure 1.** Pie graphs demonstrating and comparing the molecular pathways of both the 74 IMPC genes and the 114 gold standard genes, using the Panther function on Gene Ontology. Pathways with the red box are only implicated from the mouse data from our genes. The genes implicated within the pathways with the red box are labeled next to the pathway.

**
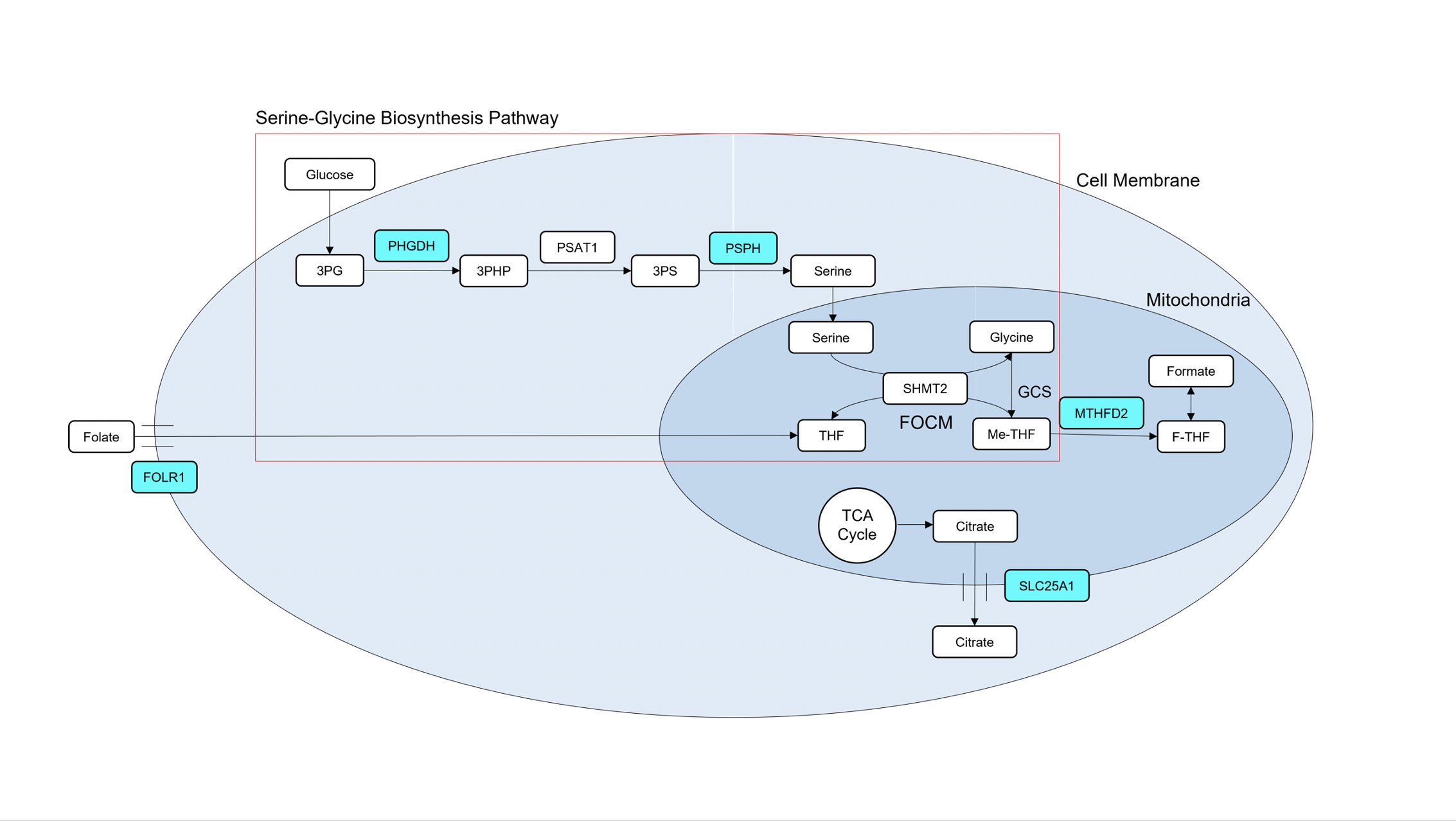
**

**Supplemental Figure 2**. Serine-Glycine biosynthesis pathway derived from the KEGG pathway on DAVID, based on pathways and gene interactions predicted by gene ontology and STRING analysis. IMPC genes with MAC phenotypes (blue) are highlighted.

**
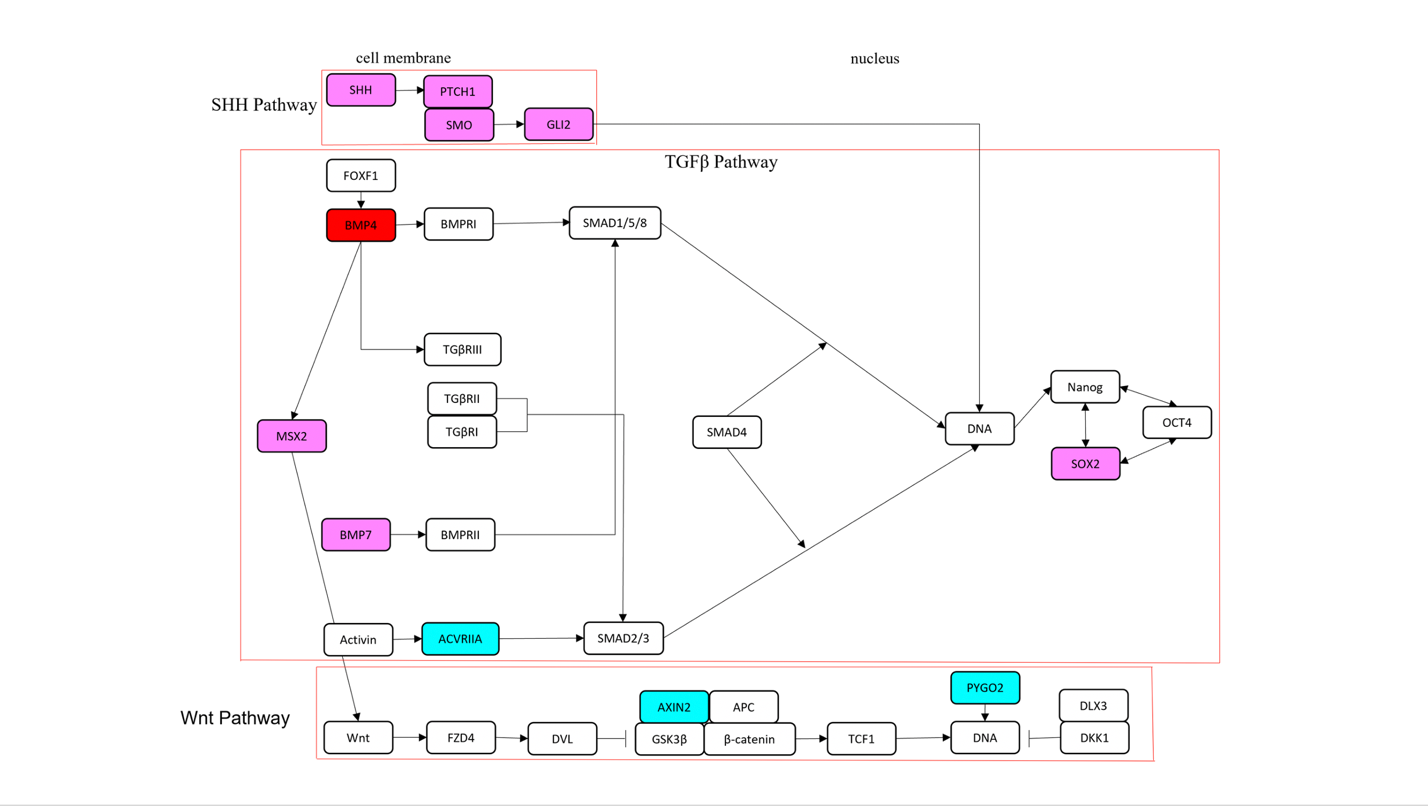
**

**Supplemental Figure 3**. Interrelationships between pathways involved in stem cell maintenance and proliferation derived from the KEGG pathway on DAVID. Established MAC genes (pink) and IMPC genes with MAC phenotypes (blue) are highlighted. Red indicates a gene in both groups, and SOX2 is highlighted (pink) as an established critical MAC spectrum disease gene.


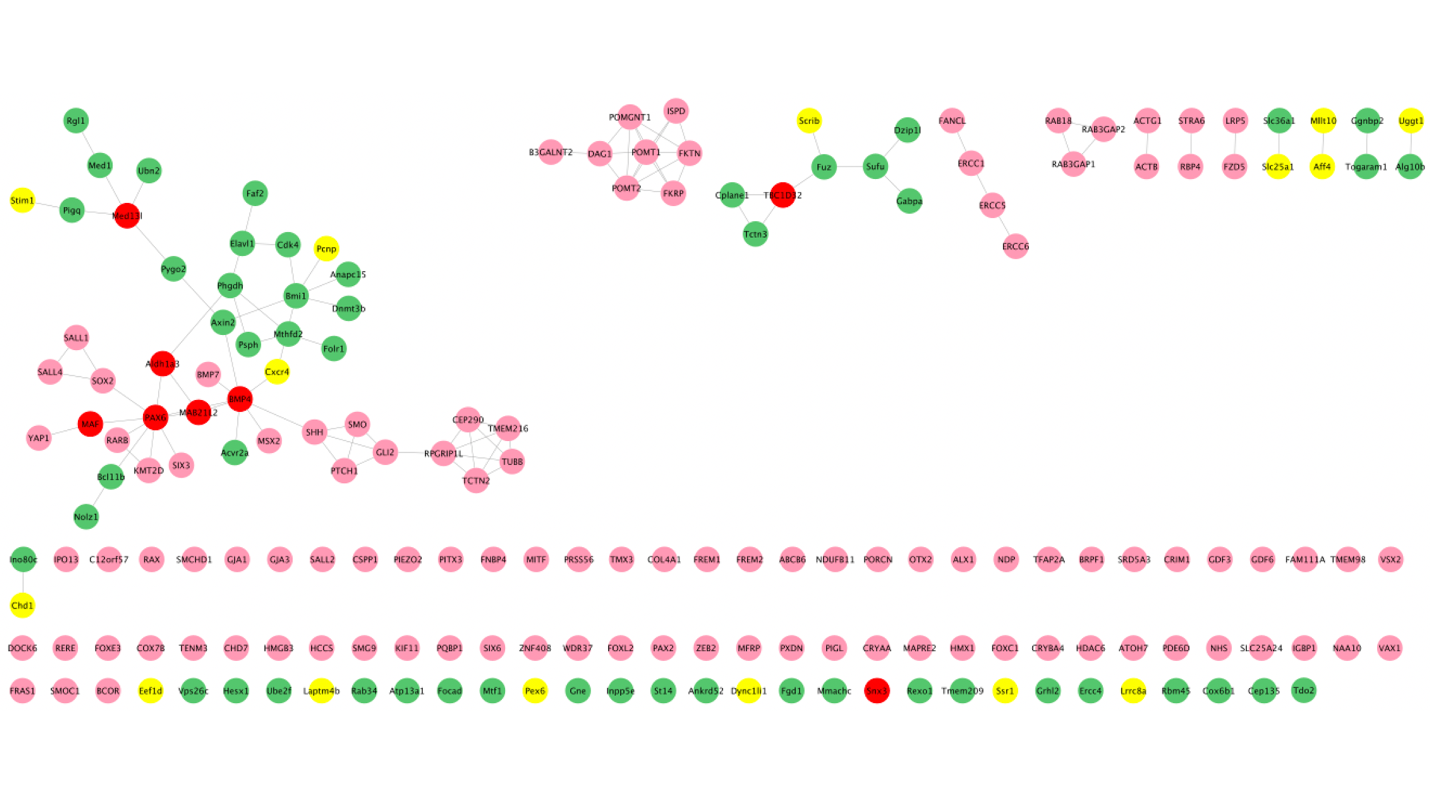
**Supplemental Figure 4.** Analysis of protein-protein interactions between the IMPC genes and the Gold Standard genes within a merged network. Cytoscape was the software platform used to merge the two networks together, and the protein interactions between the two groups of genes were analyzed using the STRING biological database software program. The arrows point towards genes that are initially not incorporated into STRING clusters until merged together within the Cytoscape platform.

**
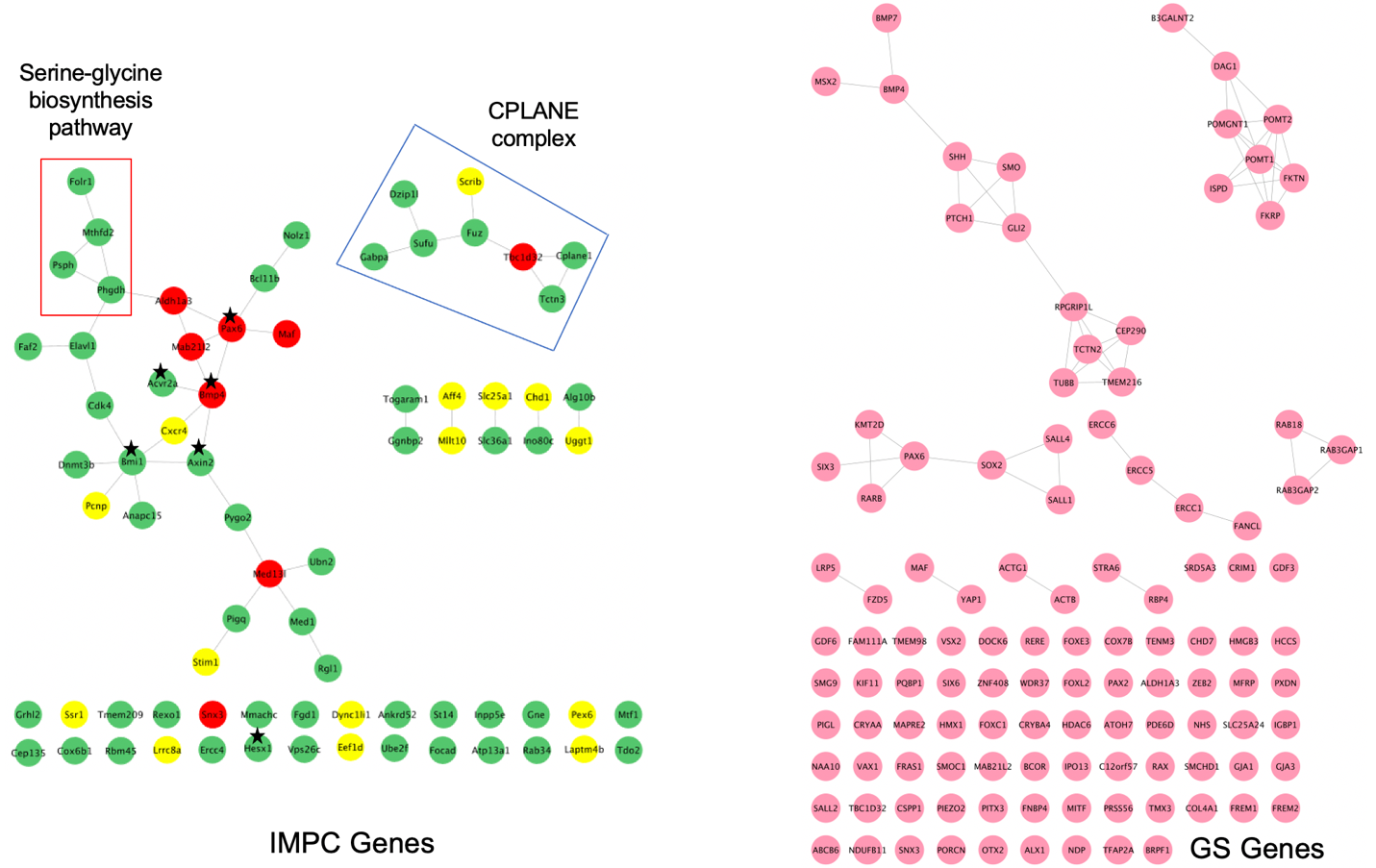
**

**Supplemental Figure 5.** Analysis of protein-protein interactions in the 74 IMPC genes (left) and the 114 Gold Standard genes (pink, right) using STRING biological database software. Among the IMPC genes, strongly significant (P<0.0001, green) and conventionally significant (P<0.05, yellow) genes are shown. Red genes are those that also appear on the list of gold standard MAC genes. Genes with stars (n=6) are members of signaling pathways regulating pluripotency of stem cells. Clusters encircled represent the serine-glycine pathway (red box) and Cplane1 complex (blue box).

**Supplemental Tables**

***All Supplemental Tables are individually uploaded as an Excel file.

**Supplemental Table 1.** Knockout mice exhibiting phenotypic embryonic eye defects. The embryonic eye defects were divided into four categories: Abnormal eyelid fusion, abnormal optic vesicle formation, microphthalmia, and anophthalmia. If a knockout strain of the gene has already been created, a PMID is listed, along with a description of the ocular defect, age, and zygosity of the mice upon diagnosis if pertinent. If there is an ocular defect present within humans (Human Eye PMID), or if there is a weak, potential correlation between the gene and an ocular defect in any species besides mice and humans (Associated Phenotypes), the PMID is listed as well. Any systemic co-phenotypes (numerical code listed below) associated with either homozygous or heterozygous mice are listed, along with the description of the heterozygous eye phenotype. From the IMPC database, if a photo of ocular defects was present in heterozygous adult mice, the type of photo taken was described in the spreadsheet. Additionally, from the same database, the phenotypic embryonic eye defects were labelled for each knockout strain, along with the age the mice were diagnosed with the defect (E9.5, E12.5, E15.5, E18.5), their homozygous viability, the zygosity of the mice with the phenotypic eye defect, and a ratio of mutant KO to normal KO mice by sex. In conclusion, the IMPC yielded 74 genes that created knockout strains with an embryonic eye defect, 27 of which had already had previously published mouse knockouts, but only15 of these knockouts exhibiting an eye abnormality. There are a total of 59 genes that are associated with embryonic eye defects in mice of which 19 genes had a previously reported human eye phenotype. Therefore, there are a total of 40 genes not previously associated with eye defects; these are bolded and highlighted in red in the first column of the table.

1 = Endocrine/exocrine                          11 = Respiratory

2 = Taste/olfaction                   12 = Cardiovascular

3 = Ear, hearing, vestibular                    13 = Behavior, Neurologic

4 = Craniofacial                                      14 = Metabolism/homeostasis

5 = Muscle phenotype                            15 = Growth and Body Size

6 = Immune/Hematopoietic                    16 = Reproductive System

7 = Skeletal Phenotype                          17 = Embryonic

8 = Limbs, digits, tail                              18 = Mortality, aging

9 = Integumentary/Pigmentation           19 = Eyes

10 = Digestive 20 = Urinary

**Supplemental Table 2**. The Gold standard list of 114 genes was created from a list of previously published genes that contribute to MAC spectrum disorders in humans. The 74 IMPC list of genes is listed beside it, and the genes present on both the gold standard list and IMPC list are highlighted in yellow. References from which the gold standard list was curated from are listed on the column to the furthest right (PMID or DOI).

**Supplemental Table 3**. Genes implicated in ciliopathies from both the IMPC and Gold Standard list of genes. The genes on column A represent IMPC genes that are implicated in ciliopathies, while the genes in column F represent Gold Standard genes that are implicated in ciliopathies.

**SI References**

Ashburner M, Ball CA, Blake JA, et al. Gene ontology: tool for the unification of biology. The Gene Ontology Consortium. *Nat Genet*. 2000;25(1):25-29. doi:10.1038/75556

Blake, J. A. *et al.* (2015) ‘Gene ontology consortium: Going forward’, *Nucleic Acids Research*. doi: 10.1093/nar/gku1179.

Brown, S. D. M. and Moore, M. W. (2012a) ‘The International Mouse Phenotyping Consortium: Past and future perspectives on mouse phenotyping’, *Mammalian Genome*. doi: 10.1007/s00335-012-9427-x.

Brown, S. D. M. and Moore, M. W. (2012b) ‘Towards an encyclopaedia of mammalian gene function: The International Mouse Phenotyping Consortium’, *DMM Disease Models and Mechanisms*. doi: 10.1242/dmm.009878.

Gene Ontology Consortium. The Gene Ontology resource: enriching a GOld mine. *Nucleic Acids Res*. 2021;49(D1):D325-D334. doi:10.1093/nar/gkaa1113

Huang, D. W. *et al.* (2007) ‘DAVID Bioinformatics Resources: Expanded annotation database and novel algorithms to better extract biology from large gene lists’, *Nucleic Acids Research*. doi: 10.1093/nar/gkm415.

Kanehisa, M. and Goto, S. (2000) ‘KEGG: Kyoto Encyclopedia of Genes and Genomes’, *Nucleic Acids Research*. doi: 10.1093/nar/28.1.27.

Koscielny, G. *et al.* (2014) ‘The International Mouse Phenotyping Consortium Web Portal, a unified point of access for knockout mice and related phenotyping data’, *Nucleic Acids Research*. doi: 10.1093/nar/gkt977.

Mi, H. *et al.* (2017) ‘PANTHER version 11: Expanded annotation data from Gene Ontology and Reactome pathways, and data analysis tool enhancements’, *Nucleic Acids Research*. doi: 10.1093/nar/gkw1138.

Szklarczyk D, Franceschini A, Wyder S, et al. STRING v10: protein-protein interaction networks, integrated over the tree of life. *Nucleic Acids Res*. 2015;43(Database issue):D447-D452. doi:10.1093/nar/gku1003
